# Phylogenetic tree of Litopterna and Perissodactyla indicates a complex early history of hoofed mammals

**DOI:** 10.1038/s41598-020-70287-5

**Published:** 2020-08-06

**Authors:** Nicolás R. Chimento, Federico L. Agnolin

**Affiliations:** 1 Laboratorio de Anatomía Comparada y Evolución de los Vertebrados (LACEV) Museo Argentino de Ciencias Naturales “Bernardino Rivadavia” (MACN), Av. Ángel Gallardo 470 (C1405DJR), Buenos Aires, Argentina; 2 Consejo Nacional de Investigaciones Científicas y Técnicas (CONICET), Buenos Aires, Argentina; 3 Fundación de Historia Natural “Félix de Azara”, Centro de Ciencias Naturales, Ambientales y Antropológicas, Universidad Maimónides, Hidalgo 775 (C1405BDB), Buenos Aires, Argentina

**Keywords:** Palaeontology, Palaeontology, Phylogenetics

## Abstract

The Litopterna is an extinct clade of endemic South American ungulates that range from Paleocene up to late Pleistocene times. Because of their unique anatomy, litopterns are of uncertain phylogenetic affinities. However, some nineteenth century authors, considered litopterns as related to perissodactyl ungulates, a hypothesis recently sustained by molecular data. The aim of the present contribution is to include litopterns and other South American related taxa in a comprehensive phylogenetic analysis together with several extant and extinct basal perissodactyl ungulates. The analysis resulted in the nesting of litopterns and kin as successive stem-clades of crown Perissodactyla. Further, litopterns are not phylogenetically grouped with any North American basal ungulate, in agreement with some previous proposals. Presence of pan-perissodactyls in South America and India indicates that southern continents probably played an important role in the early evolution of hoofed mammals.

## Introduction

The mammalian group Litopterna was coined by Ameghino^1^ as a Suborder of the Perissodactyla, with the aim to include the aberrant *Macrauchenia* and its kin.

Ameghino recognized affinities with the Laurasian clade Perissodactyla, a hypothesis sustained by some old workers^2,3^. This idea was posteriorly criticized and refuted, and it was proposed that the similarities between litopterns and perissodactyls were acquired by convergence^4,5^. In the same line of thought, together with xenarthrans and marsupials, South American native ungulates were considered by Simpson^6,7^ as comprising the “Ancient Immigrants” Faunistic Stratum, coming from North America through a intercontinental bridge. Since then, the Litopterna weas regarded as an endemic clade exclusive of South America, with uncertain affinities to other mammalian lineages. In line with Simpson proposal, most authors indicate that litopterns were the descendants of “ancient ungulates” arriving at South America from North America by a land connection at the Latest Cretaceous–Early Paleocene^8–10^.

Recent phylogenetic analysis based on protein spectrometry and DNA analyses resulted in the referral of Litopterna to Perissodactyla^11–13^, in agreement with nineteenth century authors. The aim of the present work is to include representatives of Litopterna within a comprehensive morphological data matrix of basal ungulates and to test, on the basis of morphology, the phylogenetic results obtained by previous authors^12^, as well as to discuss the palaeobiogeographical implications of litoptern affinities.

## Results

**Figure 1:**
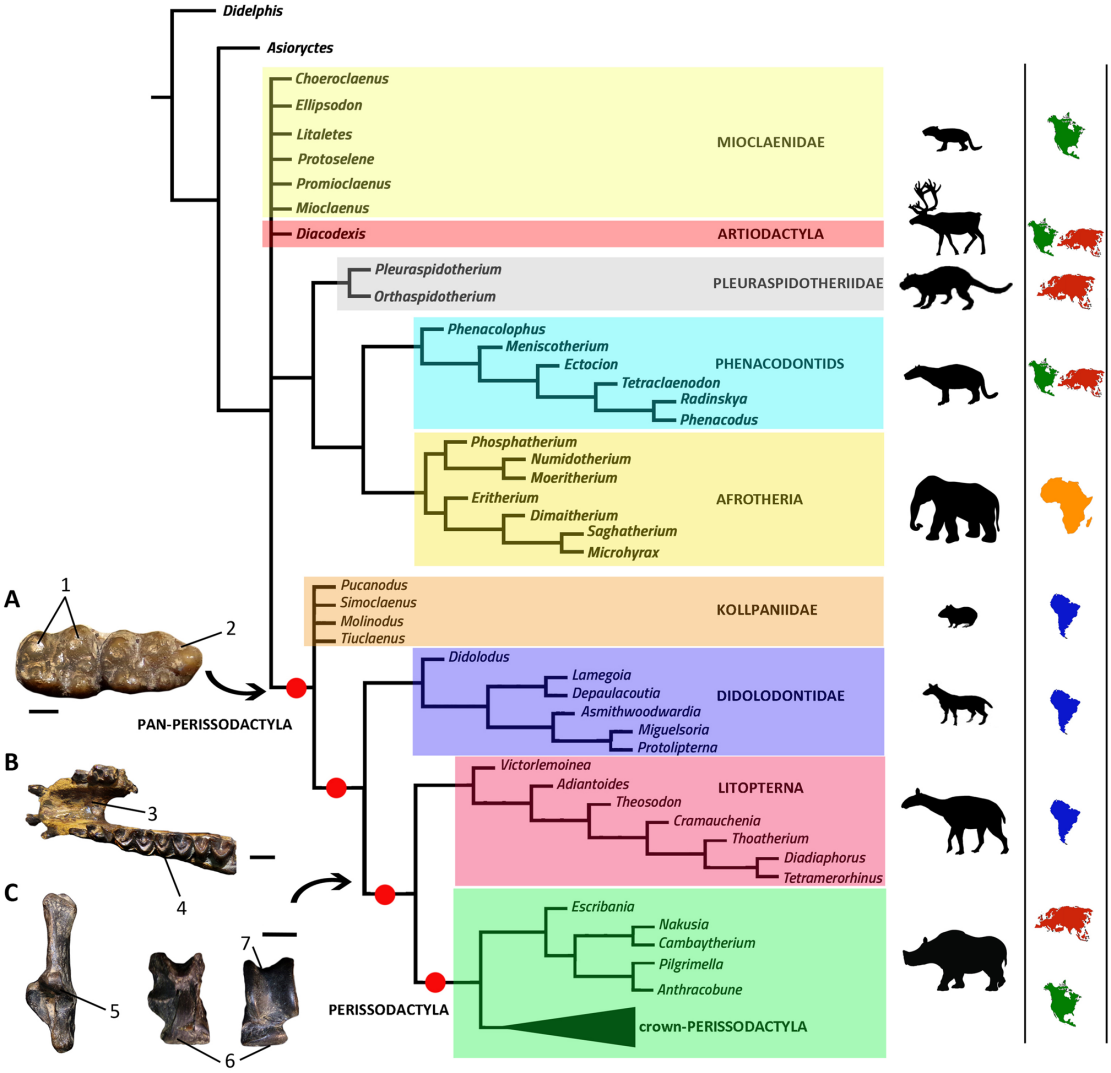
Simplified cladogram showing key anatomical traits in pan-Perissodactyla tree. (**A**) right m2-3 of *Didolodus multicuspis* (MACN A-10689) in occlusal view; (**B**) lower jaw with left p3-m2 of *Thoatherium minusculum* (MACN A-2980-89); (**C**) left calcaneum (posterior view) of *Thoatherium minusculum* (MACN A-2980-89) and left astragalus (ventral and dorsal views) of *Tetramerorhinus mixtum* (MACN A-3009-3015). Abbreviations: 1, bulbous lower molars with apices of cusps approximated to each other; 2, well-defined third lobe on lower m3; 3, fused symphysis; 4, selenodont lower molars; 5, posterior astragalar facet of the calcaneum angular and interlocks with the astragalus; 6, saddle-shaped navicular facet of astragalus; 7, narrow and deep astragalar trochlea. Scale bar: **A**–**C**, 5 mm.

Phylogenetic analysis here performed is congruent with recent claims, based on molecular evidence, in which Litopterna is nested within Pan-Perissodactyla, as the sister group of remaining perissodactyls^11–13^ (Fig. 1). The inclusion of Litopterna among perissodactyls partially returns to the old ideas of Ameghino^14^. However, in contrast with the last author, and in agreement with Cifelli^15^, we also consider Didolodontidae as closely related to litopterns.

**Figure 2:**
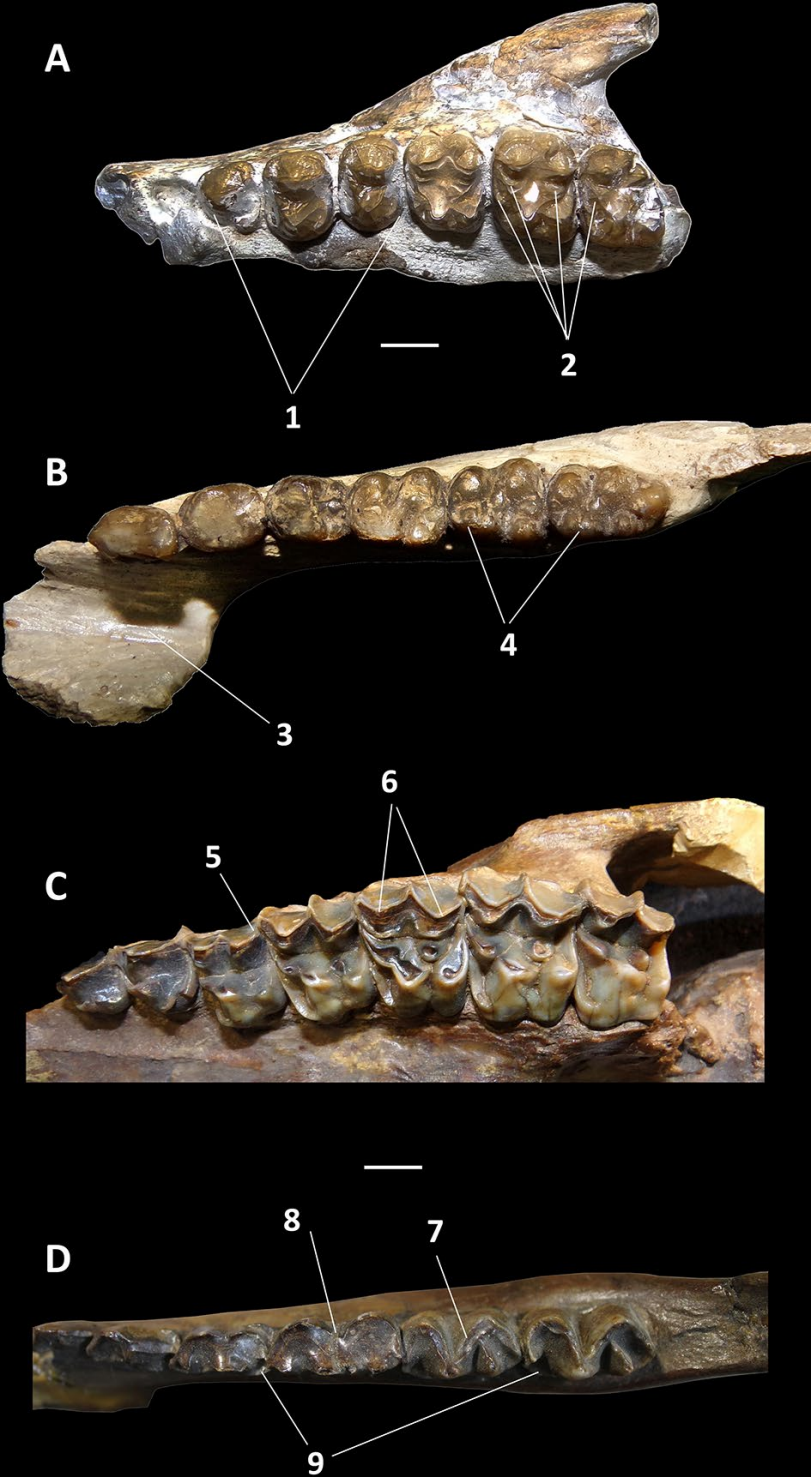
Dentition of didolodontid and litoptern ungulates, showing selected phylogenetically informative traits. (**A**,**B**) *Didolodus multicuspis*, (**A**) left maxilla with P3-M3 in occlusal view (MACN A-10690), (**B**) right dentary with p2-m3 in occlusal view (MACN A-10689); (**C**) *Tetramerorhinus mixtum* left upper P1-M3 in occlusal view (MACN A-8970/98, holotype); (**D**) *Theosodon glacilis* right lower jaw with p3-m3 in occlusal view (MACN A-9269/88). Abbreviations: 1, non-molariform premolars; 2, additional conules; 3, fused dentary symphysis; 4, twinned metaconids; 5, prominent parastyle; 6, paracone and metacone subequal in size and shape; 7, well-developed cristid obliqua; 8, reduced valley between talonid and trigonid; 9, well developed lingual crests. Scale bar: (**A**,**B**) 5 mm; (**C**,**D**), 1 cm.

The analysis resulted in that Kollpaniidae, Didolodontidae, and Litopterna form successive stem-groups to Perissodactyla. All these taxa are united by features commonly regarded as diagnostic of perissodactyls, including metacone on P3 present but smaller than paracone (character 118, state 1), p3 metaconid present and close to protoconid (character 174, state 1), p4 entoconid absent (character 182, state 0), and m2 hypoconulid separate from hypolophid (character 203, state 0) (Figs. 1 and 2). This combination of characters is present in most known pan-perissodactyls, and sustains the perissodactyl affinities of litopterns, and South American “condylarths”. It is worthy to mention that such combination of characters is totally absent in North American Paleogene Mioclaenidae “condylarths”, such as *Mioclaenus* and *Promioclaenus*^16,17^. These have been considered the group that most likely gave rise to the South American “condylarths” and litopterns^15,18,19^. Further, kollpaniids as *Molinodus, Simoclaenus* and *Tiuclaenus* differ from typical mioclaenids as *Promioclaenus*, and resemble didolodontids, basal litopterns and perissodactyls in having more bulbous lower molars, with apices of the cusps more approximated, in the longer trigonid of lower molars with paraconid more separated from metaconid, in the enlarged m3 and in the unreduced M3^20^ (Figs. 1 and 2).

Dental similarities between South American condylarths and litopterns were previously noted by several authors, whom indicate that they may form a monophyletic clade^4,8,16,18,20^, for which the name Panameriungulata is available. Present results partially agree with such proposal, being congruent in that South American condylarths and litopterns constitute successive stem-taxa of Perissodactyla.

South American condylarths have been variously allied to the North American families Arctocyonidae, Hyopsodontidae, Phenacodontidae, Periptychidae, and Mioclaenidae^8,15,17,19–24^. Cifelli^15^ suggested that North American mioclaenines could serve as structural ancestors for the South American Didolodontidae, and numerous workers sustained a close relationship between North American Mioclaenidae and South American ungulates^18,20–25^. However, it has recently been remarked that there is no support of close phylogenetic relationships between North American Mioclaenidae and South American condylarths and native ungulates. Even detailed morphological analysis did not find any derived character shared between Mioclaenidae and South American or African taxa^26^.

In sum, present analysis indicates that South American condylarths are probably not closely allied to Northern Hemisphere taxa. As indicated above, South American forms share a number of derived features with perissodactyls that are absent in basal North American ungulate taxa.

The monophyly of Kollpaniidae resulted unresolved, with *Pucanodus, Molinodus, Simoclaenus* and *Tiuclaenus,* conforming a basal polytomy to remaining Pan-Perissodactyla. Because it is not the aim of the present analysis to resolve the internal relationships among kollpaniids, we do not discuss the monophyly of this grouping in length.

*Miguelsoria* and *Protolipterna* were first included as belonging to Protolipternidae^15,19^. Here they are included in the Didolodontidae, following recent proposals^27^. The clade including Didolodontidae + (Litopterna + Perissodactyla) is sustained by six unambiguous synapomorphies, namely: P4 with metacone subequal in size to paracone (character 126, state 1), M3 size subequal or larger than M2 (character 161, states 1–2), M3 metacone lingually shifted (character 162, state 1), lingual metaconid buttress on lower molars (character 188, state 1), buccally tilted paracone on upper molars (character 211, state 1), and lower molars hypoconid large, extending on the lingual half of the talonid, invading talonid basin anterior to hypoconulid (character 213, state 1). Characters 161, 188, 211, and 213, are features typically considered as diagnostic of Perissodactyla^28,29^, and were regarded as widespread among didolodontids, such as *Didolodus* and *Asmithwoodwardia*^30,31^, as well as litopterns (e.g., *Proterotherium, Victorlemoinea*^4,15^), and are also observed in *Escribania*^10,24^. These traits are totally absent in other basal ungulates including South American “condylarths” of the clade Kollpaniidae^18,20^.

In addition to the above mentioned synapomorphies, some other key-traits shared by didolodontids, litopterns and perissodactyls include a fused mandibular symphysis, twinned lower molar metaconids, and a well-defined third lobe on the last lower molar, a combination of traits previously considered as unique to perissodactyls^32–35^ (Fig. 2). Didolodontidae shares with basal perissodactyls as cambaytheriids and anthracobunids many plesiomorphic features including bunodont cheek-teeth with well-developed conules on upper molars, and the lack of any hint of lophodonty^35^. In fact, very prominent conules are usually considered to be diagnostic of didolodontids^16,27^, but are present also in cambaytheriids and anthracobunids^34–36^, sustaining close relationships between these clades.

**Figure 3:**
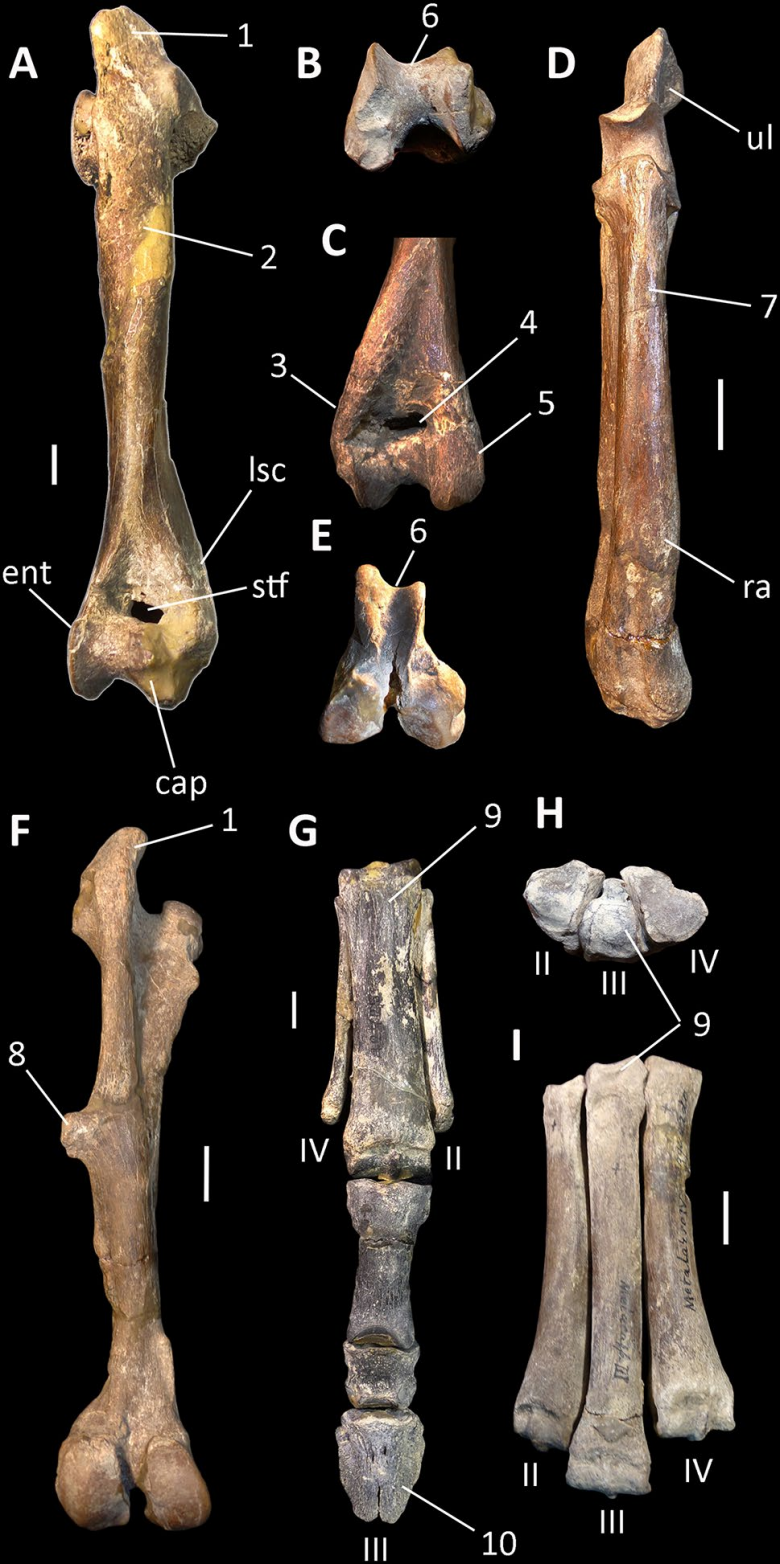
Selected postcranial elements of litopterns. (**A**–**F**) *Tetramerorhinus mixtum* (MACN A-8970/98), (**A**–**C**) right humerus in (**A**) anterior; (**B**) distal; and (**C**) posterior views; (**D**) right radius and ulna in anterior view; (**E**,**F**) left femur in (**E**) distal, and (**F**) posterior views; (**G**) *Diadiaphorus majusculus* (MACN A-2713/37) right foot in anterior view; (**H**,**I**) *Theosodon lyddekeri* (MACN A-11027) left foot in (**H**) proximal, and (**I**) anterior views. *cap* capitulum, *ent* entepicondyle, *lsc* lateral supinator crest, *ra* radius, *stf* supratrochlear foramen, *ul* ulna, 1, prominent greater trochanter; 2, not prominent and proximally restricted deltopectoral crest; 3, reduced lateral supinator crest; 4, wide and deep supratrochlear foramen; 5, reduced entepicondyle; 6, transversely narrow trochlea delimited by acute ridges; 7, radius anterior to ulna; 8, prominent and large third trochanter; 9, mesaxonic foot; hoof-like ungual phalanges. Scale bar: (**A**–**E**) 1 cm; (**F**–**I**) 2 cm.

**Figure 4:**
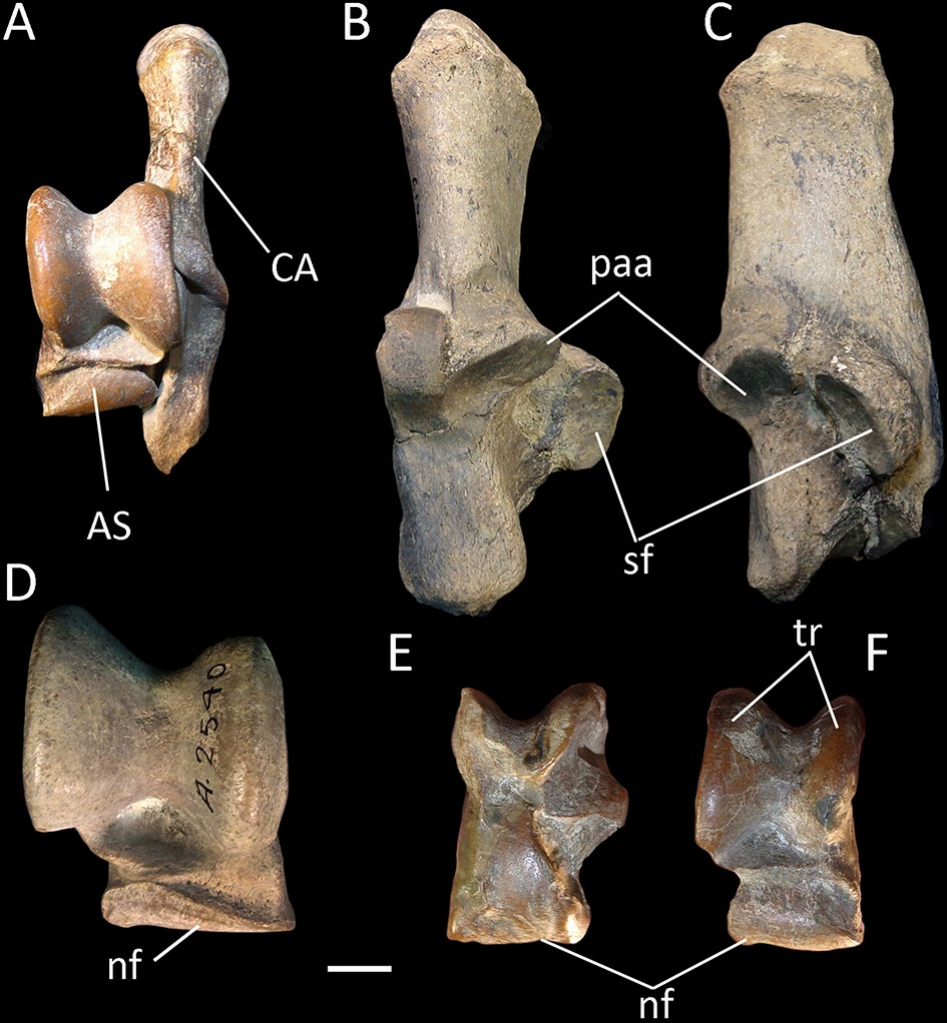
Tarsal bones of selected litopterns. (**A**,**E**,**F**) *Tetramerorhinus mixtum*; (**A**) (MACN A-8970/98) left articulated calcaneum and astragalus in dorsal view; (**B**,**C**) *Theosodon lyddekeri* (MACN A-2619–24) right calcaneum in (**B**) dorsal, and (**C**) medial views; (**D**) *Theosodon lyddekeri* (MACN A-10977/78) right astragalus in dorsal view; (**E**,**F**) right astragalus in (**E**) ventral and (**F**) dorsal views. *AS* astragalus, *CA* calcaneum, *nf* navicular facet, *paa* posterior astragalar articulation, *sf* sustentacular facet, *tr* astragalar trochlea. Scale bar: (**A**,**E**,**F**) 5 mm; (**B**–**D**) 1 cm.

Litopterns and perissodactyls share a number of apomorphies absent in basal ungulates and all South American “condylarths”, including didolodontids. These traits include a saddle-shaped navicular facet of astragalus (character 86, state 1), P3 parastyle protruding, with mesial edge concave (character 115, state 0), paracone and metacone of M1-2 about the same size (character 154, state 1), p4 paralophid well developed without paraconid, and mesially directed (character 179, state 2), m1 paralophid extending lingually and connected to mesial crest from metaconid (character 183, state 3), well-developed lower molar cristid obliqua obliquely oriented and contacting lingual cusps (character 191, state 1), resulting in a reduced valley between trigonid and talonid (character 194, state 1), m3 hypolophid complete, lingual and labial cristids subequal in length (character 196, state 1), lower molar posthypocristid absent (character 198, state 1), and m2 hypoconulid closely appressed to hypolophid (character 203, state 1). Most of the listed dental traits are related with the rearrangement of cusps due to the development of cristids and lophids, resulting in the progressive acquisition of selenodont dentition characterizing perissodactyls and litopterns^28,29,34,37^ (Figs. 2 and 3). Presence of saddle-shaped navicular facet of astragalus was recently regarded as one of the key-characters diagnosing Perissodactyla^35,38^ (Figs. 2, 3 and 4). Regarding the latter feature, it appears that the didolodontids had a primitive-like astragali^15,39^, showing an homogeneously convex navicular facet, very different from the saddle-shaped morphology reported for litopterns and perissodactyls^4,19,35^.

In addition, litopterns share a large number of postcranial traits previously regarded as typical of Perissodactyla, including mesaxonic foot symmetry with reduced metapodials I and V, and hoof-like terminal phalanges, femur with large third trochanter and prominent greater trochanter, and very expanded greater trochanter on humerus (much more expanded than in basal condylarths as *Phenacodus, Arctocyon* or *Tetraclaenodon*^40,41^) (Fig. 3), the distal humeral articulation is strikingly narrow and high, proximally delimited by a large foramen, and the radius is anteriorly located to the ulna. These features are correlated with an increased stride length and joints with reduced rotation^1,35,42^, a combination of characters typical of perissodactyls^35^.

In litopterns, as occurs in perissodactyls, the deltopectoral crest of humerus is not protrudent, and is restriced to the proximal half of the bone, whereas in phenacodontids and cambaytheriids the crest is distinct and plesiomorphically extends towards the distal end of the bone^35^. Further, the entepicondyles and the lateral supinator crest are reduced, contrasting with condylarths and basal perissodactyls as cambaytheriids^35,40,41^. In addition, the posterior astragalar facet of the calcaneum is angular and interlocks with the atragalus, whereas in cambaytheriids and condylarths this facet is rounded^35^.

**Figure 5:**
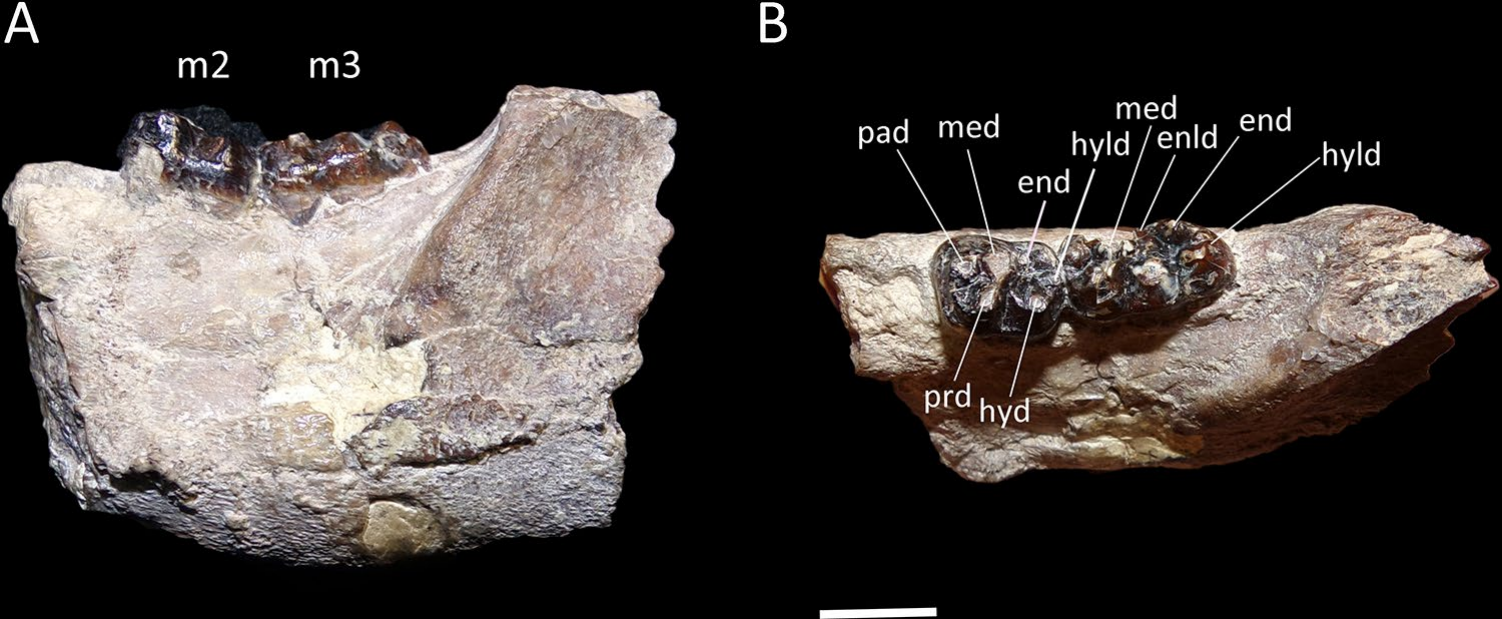
*Escribania chubutensis* (UNPSJB PV 916, holotype). Posterior portion of left dentary with m2-3, in (**A**) lateral, and (**B**) occlusal views. *enld* entoconulid, *end* entoconid, *hyld* hypoconulid, *hyd* hypoconid, *med* metaconid, *pad* paraconid, *prd* protoconid. Scale bar: 5 mm.

One surprising result of present analysis was the nesting of the South American condylarth *Escribania* among Paleogene Indian Cambaytheriidae and Anthracobunidae. These taxa share some unambiguous synapomorphies, including absence of lower molar metaconid buttress (character 188, state 0), individualized protostyle on upper molars (character 212, state 1), and distinct entoconulid on lower molars (character 207, state 0) (Fig. 5). We here interpret the large and well-developed cusp in the lower molars of *Escribania*, and described as the “accesory cusp 2” by Gelfo et al.^10^ as the entoconulid, because it is located anteromedially to the entoconid cusp.

*Escribania* shares with didolodontids, litopterns and perissodactyls several features (e.g., m3 with entoconid similar in size to hypoconulid, entoconid and hypoconulid separate, absence of entocristid, and presence of additional cusp mesial to entoconid). However, it differs from didolodontids in several dental traits: m3 with inflated metaconid that invades the talonid basin, relatively narrower talonid, and large trigonid with well-developed paraconid^10,24^. Further, *Escribania* shows a large parastyle as large as the mesostyle^10^. These features are clearly present in cambaytheriids, such as *Cambaytherium*^35,43^.

Perissodactyls sensu stricto, excluding litopterns, and South American condylarths are joined by a large combination apomorphies: absence of first metacarpal (character 63, state 1), metaconule mesially displaced on P4 (character 128, state 2), preparaconule crista on upper molars joined with paracone (character 155, state 2), and m3 hypoconulid connection joining mid-hypolophid (character 206, state 1), among others.

Cambaytheriidae and Anthracobunidae result included in the sister-group of remaining Perissodactyla, in agreement with recent contributions^35,36^.

## Discussion

Recently, on the basis of protein analysis, Welker et al.^12^ suggested that notoungulates and litopterns may belong to Perissodactyla. Regarding notoungulates, many authors indicate that they are probably not phylogenetically close to litopterns^18^, and that notoungulates share features with afrotherians^44,45^. This last proposal resulted in a hot debate about notoungulate origins^46,47^. In this way, present discussion will focus on the biogeographic implications of perissodactyl affinities for litopterns.

### Litoptern affinities and the splendid isolation of South America

Seminal studies by Florentino Ameghino on fossil mammals from Patagonia resulted in a number of biogeographical relationships for the entire mammalian clade. This paleontologist^14^, proposed that most mammals originate in the Southern Cone and from there dispersed trough the entire world, a point of view known as “Extreme Australism”^48^. This was refuted by Albert Gaudry^49^ who considered that most characters linking Argentinean fossils with those of other landmasses are the result of convergences through a long time of isolated and parallel evolution, a “Splendid Isolation” as coined by Simpson^50^.

In spite that most authors (with exception of Muizon and Cifelli^18^) were not able to find special similarities between North American and South American basal ungulates, it was clear to them that South American Condylarths undoubtedly arrived from North America^8,11^. Present work failed to find a clade encompassing South American and North American Condylarths, suggesting the possibility that South American litopterns may not be necessarily related to Northern Hemisphere taxa, in agreement with some previous authors^26^.

In this sense, the model of South America isolation may be too biotically simplistic^51^, as demonstrated by several studies which indicate that several animal and plant lineages reached South America from Africa by Late Cretaceous and Tertiary (e.g., legumes, lauraceans, and several others^52^). On this basis, authors indicate that Africa and South America may have been united by Walvis Ridge-Río Grande Rise, and Sierra Leone-Ceará Rises during the Early Tertiary^53–55^. This is sustained by a large number of taxa shared between Africa and South America, but also with other landmasses and especially India, including hystricognath rodents, anthropoid monkeys, afrotherian mammals, pipid frogs, freshwater fishes (cichlids and aplocheiloids), birds (parrots, hoatzins, phororhacoids), and lizards (geckos), and Malpighiaceae, Asteraceae, and Bromeliaceae among plants^52,54–59^. Further support for this interchange includes the finding of several lineages of metatherians, anthropoid monkeys and hystricognath rodents in South America, indicating multiple dispersals between South America and Africa and vice-versa during the Paleogene^60–64^. As enumerated above, the evidence indicating a fluid interchange between South America and other Southern Hemisphere landmasses and India has been greatly increasing during the last years (see below). This is in agreement with the seminal idea of Lavocat^65^ whom suggested that the fossil record indicates closer biogeographical ties between South America and Africa than between North and South America.

As summarized above, strong biotic connection between South America and former Gondwanan landmasses appears to come to light. This point is crucial for understanding early biogeographical relationships of mammals, and more efforts are urgently need in order to analyze and criticize in detail different biogeographical scenarios.

### India-South America biogeographical relationships

As indicated above, there are striking similarities between the Latest Cretaceous and Paleogene faunas and floras of former Gondwanan continents, including South America, Africa, and India. Bonaparte^66^ noted that Mesozoic faunas from India were undoubtely Gondwanan in origin. In contrast, authors agree that the collision of India with Asia during the latest Cretaceous or Paleogene resulted in an important faunistic exchange^67,68^, and conclude that Paleogene faunas from India were entirely composed by Laurasian taxa^69,70^.

However, some recent workers sustained an important influence of Gondwanan biogeographical ties on India up to the early Tertiary. New findings suggest that by Eocene times Indian faunas were “mixed”, having both European and Gondwanan lineages. Typically Gondwanan taxa include madtsoiid snakes, dyrosaurid crocodiles and pelomedusoid turtles^43^. More recently, adapisoriculid mammals with strong Gondwanan ties were reported for the first time in the early Eocene of India^71,72^.

Present analysis resulted in the shared presence of basal perissodactyls in both India and South America (Fig. 1). Further, the genus *Escribania* was included as the sister group of the Indian clade Cambaytheriidae + Anthracobunidae. In this way, perissodactyls constitute another clade that adds to the list of taxa shared by India and South America. It is possible that as soon as the fossil record of Paleogene faunas of India becomes improved, the list of taxa shared by both landmasses might increase.

Smith et al.^43^ summarized two main hypotheses explaining occurrence of Gondwanan faunas on India. The first hypothesis proposed that these Gondwanan taxa may be the descendants of taxa already present by Cretaceous times that survived the K/T boundary. The second hypothesis sustain that a dispersal of Gondwanan taxa occurred from North Africa along the margins of the Neotethys to India. In this regard, an island arch (Oman–Kohistan–Dras) has been the route of migration proposed between Africa and India, during the Latest Cretaceous^71–74^. Because of the meagre fossil record, both hypotheses still lack important empiric support. However, because perissodactyls lack Cretaceous records, the shared presence of these taxa in both South America and India (and possibly Africa) may indicate Early Tertiary dispersal of Gondwanan taxa between India and North Africa.

### Origin and early radiation of Perissodactyla

The first works that deal with the origin of hoofed mammals indicate an Holarctic craddle for the Perissodactyla, particularly North American^29,75,76^ or Asiatic origins^36^.

However, in the last decades many authors proposed that perissodactyls may have originated on India prior to its collision with Asia. Under this hypothesis the Indian plate may have acted as a “Noah´s Ark” during the Cretaceous and Paleocene^73^. Then, India carried Gondwanan forms to Asia after the break-up of the Gondwana super continent. This “Out of India” model was followed with modifications by some authors whom sustained that Indo-Pakistan area was most likely the center of origin for the Perissodactyls^35,38,74^. Further, Rose et al.^35^ suggested that stem-Perissodactyla could have dispersed to India from Africa, by early Paleocene, and then, given rise to Perissodactyla before contact of India with Asia. In partial agreement with these contributions, present phylogenetic analysis indicates that pan-perissodactyls were widespread on southern continents, particularly in India and South America (and possibly in Africa) by early Tertiary times. This suggests that the southern continents may have played an important role in the early evolution and radiation of hoofed mammals.

## Materials and methods

We follow the general concept and nomenclature of Litopterna and Didolodontidae of Simpson^16^ with modifications by more recent authors^10,19,27^, and the Kollpaniinae of recent authorities^18,20,24^. In the later case, with the aim to emphasize the distinctiveness of the South American “mioclaenines” we opt to use Kollpaniidae rather than Kollpaniinae.

With the aim to analyze the phylogenetic relationships of Litopterna and kin we run a phylogenetic analysis following the comprehensive data matrix confected and employed by Rose et al.^35^. As in Rose et al. article, current study is limited to ungulates and does not address the possibility of a close relationship of litopterns to several disparate placental mammals (e.g., Glires, Primates, Carnivora). This data matrix was originally composed by 208 characters and 53 taxa. To this matrix we added 26 taxa (mostly “condylarths”, litopterns, and didolodontids) and 6 characters that were employed by previous authors and that are key to dilucidate the affinities of litopterns and basal ungulatomorphs. This resulted in a matrix of 214 characters and 79 taxa (Supporting Information 1–3). In addition, the character–taxon matrix is available on MorphoBank (Project 3768).

The phylogenetic analysis was performed using TNT 1.5^77^. All characters were equally weighted and treated as unordered. The data matrices were analysed under equally weighted parsimony. A total of 1,800,000 trees was set to be retained in memory, which is the maximum number of trees possible that could be saved on the computer used for these analyses. A first search using the algorithms Sectorial Searches, Ratchet (perturbation phase stopped after 20 substitutions), and Tree Fusing (5 rounds) was conducted, performing 1,000 replications in order to find all tree islands (each replication starts from a new Wagner tree). The best tree or trees obtained at the end of the replicates were subjected to a final round of TBR (tree-branch-swapping) algorithm.

The phylogenetic analysis resulted in the recovery of 8 Most Parsimonious Trees (MPTs), of 1518 steps, with a consistency index of 0.220, and a retention index of 0.625 which are summarized using a strict consensus (Fig. 1; Supporting Information 4).

As a branch support measure, Bremer support was calculated, and as a measure of branch stability, a bootstrap resampling analysis^78^ was conducted, performing 10,000 pseudoreplicates. Bremer support was calculated after searching for suboptimal trees and not with the script that accompanies the program^79^. Both absolute and GC^80^ bootstrap frequencies are reported (Supporting Information 5).

## Supplementary information

Supplementary file1 (PDF 1347 kb)
