# Supplementary material for: Phylogenetic tree of Litopterna and Perissodactyla indicates a complex early history of hoofed mammals
Source: Sci Rep. 2020 Aug 6;10:13280. doi: 10.1038/s41598-020-70287-5 (PMC7413542; doi:10.1038/s41598-020-70287-5)

PHYLOGENETIC TREE OF LITOPTERNA AND PERISSODACTYLA INDICATES A COMPLEX EARLY HISTORY OF HOOFED MAMMALS

Nicolás R. Chimento[1] & Federico L. Agnolin[1,2]

1. **Character list**
2. **Data Matrix**
3. **Synapomorphies of selected nodes.**
4. **The eight Most Parsimonious Trees (MPTs) that resulted from the phylogenetic analysis.**
5. **Figure showing phylogenetic results, including branch support measures.**

1. **Character list**

Characters

1. Posterior nasal:
- 0. narrow, not contacting lacrimal
- 1. broad contact with frontal

2. Length of postorbital portion of skull:
- 0. shorter than preorbital portion
- 1. about equal to preorbital portion
- 2. longer than preorbital portion

3. Position of orbits:
- 0. over molars
- 1. over premolars or more anterior

4. Premaxilla:
- 0. small, ascending process contacts nasals
- 1. ascending process present, no nasal contact

5. Incisive foramen:
- 0. paired
- 1. single median

6. Tuber maxillae:
- 0. weak or absent in adult
- 1. prominent in adult

7. Orbital portion of maxilla:
- 0. separated from frontal
- 1. contacting frontal

8. Sphenopalatine foramen:
- 0. middle of orbit
- 1. near maxillary foramen

9. Contribution of ascending lamina of palatine in orbit:
- 0. forms significant part of medial orbital wall
- 1. very small or absent from medial orbital wall

10. Palatal vacuities:
- 0. present
- 1. absent

11. Facial exposure of lacrimal:
- 0. large or moderate, not contacting nasal
- 1. large or moderate, contacting nasal
- 2. small, not contacting nasal

3. absent

12. Supraorbital process:
0. absent, region over orbit does not project laterally from sagittal plane
1. present, short
2. present, long and extending ventrally

13. Supraorbital foramen or notch:
0. present
1. absent

14. Optic foramen:
0. anteriorly placed
1. posteriorly placed

15. Sphenorbital fissure:
0. separate from foramen rotundum
1. confluent with foramen rotundum

16. Alisphenoid canal (posterior opening):
0. present
1. absent

17. Posterior opening of alisphenoid canal:
0. separate from foramen ovale
1. in common depression with foramen ovale

18. Foramen ovale:
0. separate
1. confluent with middle lacerate foramen

19. Foramen ovale position:
0. anterior to glenoid fossa
1. medial to glenoid fossa

20. Orbital portion of parietal:
0. contacting alisphenoid
1. not contacting alisphenoid

21. Anterior extent of jugal and zygomatic portion of maxilla:
0. jugal extends anteriorly, forms anteroventral border of orbit
1. zygomatic portion of maxilla large, jugal more posterior and does not contribute to anteroventral border of orbit

22. Posterior extent of jugal:
0. strong, contributes to anterior portion of glenoid fossa
1. strong, extends to posterior border of glenoid fossa without contributing to articular surface
2. weak, splint-like, extends to anterior edge of glenoid fossa

23. Zygomatic process of squamosal:
0. narrow
1. laterally expanded

24. Preglenoid process:
0. absent
1. present

25. Postglenoid process:
0. facing anteriorly
1. facing anterolaterally

26. Postglenoid foramen:
0. present
1. absent

27. Posttympanic process:
0. about the same size as postglenoid process
1. shortened relative to pgp

28. Exposure of mastoid:
0. broad, posterior
1. narrow, lateral
2. absent (amastoidy)

29. Mastoid foramen:
0. present, between mastoid and occipital-supraoccipital
1. absent

30. Posttemporal (or percranial) canal:
0. present at petrosal-squamosal suture, canal continues within suture
1. absent

31. Sulcus for internal carotid artery:
0. transpromontorial, forms anteroposterior groove on promontorium
1. absent

32. Sulcus for proximal stapedial artery:
0. present, forms groove that branches from transpromontorial sulcus anteromedial to fenestrae vestibuli and cochleae
1. absent

33. Tympanic aperture of hiatus Fallopii:
0. absent
1. present

34. Foramen for ramus superior of stapedial artery:
0. present, through petrosal or petrosal squamosal suture on dorsolateral edge of epitympanic recess
1. present and anterolateral, through basioccipital

2. absent

35. Ectotympanic:
0. not attached
1. attached
2. attached and forms bulla

36. Hypoglossal foramen:
0. present
1. absent

37. Height of mandibular condyle:
0. below level of dentition
1. even with superior aspect of dentition
2. substantially superior to dentition

38. Coronoid canal of dentary:
0. absent
1. present

39. Number of thoraco-lumbar vertebrae: (modified)
0. fewer than 19
1. more than 19.

Comments. The codification of some taxa was modified from 0 to 1 as follows: *Mesohippus* (17+5=22; O'Leary et al, 2013), *Eohippus* (16/17+7=23/24; Wood et al, 2011;), *Meniscotherium* (20), *Phenacodus* (15+6=21; O'Leary et al, 2013), *Palaeotherium* (19+5=21), *Orohippus* (19+5=24), and *Protohippus* (19+6=25).

40. Number of sacral vertebrae:
0. three or fewer
1. four
2. five
3. six

41. Acromion process:
0. present
1. absent

42. Bicipittal groove of humerus:
0. simple
1. with distinct "facet"

43. Ridge from deltopectoral crest extending onto distal anterior shaft of humerus:
0. present
1. absent

44. Supinator crest of humerus:
0. well-developed and prominent
1. present but restricted to distal third of shaft

2. weak or absent

45. Lateral articular shelf:
0. absent or indistinct
1. present, tapered distally
2. present, extended distally

46. Proximolateral part of lateral articular shelf:
0. flat or convex
1. concave and elaborated

47. Entepicondylar foramen:
0. present
1. absent

48. Medial epicondyle (entepicondyle):
0. very prominent, expands medially
1. prominent but not expanded
2. weak or absent

49. Supratrochlear foramen of humerus:
0. absent
1. present

50. Capitulum of humerus:
0. round
1. gently keeled
2. distinctly, sharply keeled

51. Proximal radius:
0. with single fossa for humeral capitulum and trochlea
1. with separate fossae for capitulum and trochlea

52. Proportions of head of radius:
0. low width to depth ratio
1. high width to depth ratio

53. Lateral process of radius:
0. absent or weak
1. present

54. Articular surface of lateral process of proximal radius:
0. shallowly concave
1. shallowly convex

55. Extent of proximal ulnar facet on posterior aspect of proximal radius:
0. restricted to lateral half of face
1. extending medially as narrow strip across width of head

56. Shape of lateral process of proximal radius:

0. not beveled
1. beveled to accommodate capitular tail (lateral articular shelf)

57. Styloid process of distal radius:
0. distinct and projecting distally
1. weak or absent

58. Facets on distal radius:
0. single concave fossa
1. split into separate scaphoid and lunar fossae

59. Articular surface of distal radius:
0. restricted to distal face
1. convex extension onto distopalmar surface

60. Contact between lunar and unciform:
0. present
1. absent

61. Contact between lunar and trapezoid:
0. absent
1. present

62. Centrale:
0. present as separate ossification
1. absent or fused to scaphoid

63. First metacarpal:
0. present
1. absent

64. Fifth manual digit:
0. present with phalanges
1. present without phalanges
2. absent

65. Anterior iliac crest:
0. round
1. slightly concave or straight
2. deeply concave

66. Fovea capitis of femur:
0. centrally located
1. marginal

67. Height of greater trochanter:
0. lower than head
1. about even with head
2. higher than head

68. Orientation of lesser trochanter of femur:
0. medially
1. posteromedially

69. Size third trochanter of femur:
0. small
1. large
2. absent

70. Supracondylar fossa:
0. absent
1. present

71. Trochlear ridges of distal femur:
0. subequal
1. medial expanded with tuberosity

72. Ossified patella:
0. absent
1. present

73. Medial malleolus of tibia:
0. forms well-developed medial wall
1. prominent anteriorly, reduced and beveled posteriorly

74. Posterior process and median ridge of distal articulation of tibia:
0. absent
1. present

75. Astragalar canal:
0. present
1. absent

76. Orientation of trochlear ridges of astragalus:
0. not oblique (essentially vertical)
1. oblique

77. Depth of trochlear groove of astragalus:
0. nearly flat to concave
1. shallow groove, less than 25% of trochlea width
2. deep groove, more 25% of trochlear width

78. Distal extent of medial trochlear ridge of astragalus:
0. separate from distal edge of astragalus
1. reaching distal edge of astragalus

79. Cotylar fossa:
0. absent
1. present

80. Lateral process of astragalus:
0. small
1. large and shelf-like

81. Tuberculum mediale of astragalus:
0. absent
1. present

82. Squatting facet on dorsal side of astragalar neck:
0. absent
1. present

83. Sustentacular facet of astragalus:
0. separate from distal calcaneal and ectal facets
1. confluent with distal calcaneal facet
2. J-shaped
3. confluent with ectal facet

84. Posterior tubercle of medial trochlear facet:
0. small
1. protruding
2. extending proximomedially

85. Proximal calcaneal facet of astragalus:
0. without distoectal lappet
1. with distoectal lappet

86. Navicular facet of astragalus:
0. spherical or convex
1. saddle-shaped
2. trochleated

87. Lateral groove on calcaneum:
0. present, broad
1. absent or indistinct

88. Orientation of astragalar facet of calcaneum in lateral view:
0. sloping proximally, with no angle formed within facet
1. sloping slightly proximally, facet has a rounded angle within it
2. perpendicular to long axis, facet forms sharp angle

89. Orientation of astragalar facet of calcaneum in anterior view:
0. oriented at angle to long axis
1. oriented perpendicular to long axis
2. elongated along long axis

90. Shape of facet of sustentaculum of calcaneum:
0. round or oval
1. narrow, straight on lateral edge

91. Orientation of distal edge of calcaneum between sustentaculum and cuboid facet:
0. makes wide angle with long axis
1. makes acute angle with long axis
2. expanded into distal shelf that forms right angle

92. Shape of cuboid facet of calcaneum:
0. not crescent-shaped
1. crescent-shaped

93. Peroneal tuberosity of calcaneum:
0. large
1. present, moderate, projecting distally
2. small, indistinct, or absent

94. Anterior contact between navicular and calcaneum:
0. absent
1. present

95. Plantar process of navicular:
0. present and prominent
1. weak or absent

96. Navicular and proximal ectocuneiform facets of cuboid:
0. not confluent
1. confluent with distinct ridge

97. Entocuneiform:
0. medially placed
1. posteriorly placed
2. absent

98. Entocuneiform and mesocuneiform:
0. separate
1. fused

99. Mesocuneiform and navicular facets of entocuneiform:
0. along anterior margin
1. mesocuneiform facet posterolateral to navicular facet

100. Cuboid:
0. not contacting MT III
1. contacts MT III

101. First metatarsal:
0. present with phalanges
1. present without phalanges, medially positioned
2. small and lacking phalanges, articulating with posterior MT III
3. absent

102. Fifth metatarsal:
0. present with phalanges
1. present without phalanges
2. absent

103. Distal phalanges:
0. laterally compressed, as claws
1. dorsoventrally compressed, as hooves

104. Canine size:
0. large
1. small
2. absent

105. Postcanine diastema:
0. short
1. long
2. absent

106. Cusp relief of cheek teeth:
0. sharp, generally conical
1. tall, bunodont cusps with little or no loph development
2. low, bunodont to bunolophodont
3. well-developed lophodonty with high lophs

107. Cheek tooth enamel surface:
0. smooth
1. rugose

108. Fifth premolar:
0. present
1. absent

109. P1:
0. present with diastema
1. present without diastema
2. absent

110. Diastema posterior to P2:
0. absent
1. long (greater than P3 length)
2. short (less than or equal to P3 length)

111. P2 metacone:
0. absent
1. present, small, close to paracone
2. present, about as large as and separate from paracone

112. P2 metacone position:
0. close to paracone

1. distant from paracone

113. P2 lingual cusps:

0. none
1. one
2. two

114. P3 size:

0. smaller or nearly equal to P4
1. larger than P4

115. P3 parastyle:

0. protruding, P3 mesial edge concave
1. not protruding, P3 mesial edge convex

116. P3 paraconule:

0. absent or indistinct
1. present
2. present and lingually positioned

117. P3 preparaconule crista:

0. in line with connection to protocone
1. angled more labially than connection to protocone

118. P3 metacone:

0. absent
1. present, much smaller than paracone
2. present, comparable in size to paracone

119. P3 metaconule:

0. absent or indistinct
1. present, small relative to paraconule
2. present, similar in size to paraconule

120. P3 metaloph:

0. none, metaconule separate from ectoloph and protocone
1. metaconule connected to protocone but not to ectoloph
2. metaconule connects to ectoloph but not to protocone
3. metaloph complete but weak
4. metaloph complete and prominent

121. P3 protocone:

0. absent
1. present

122. P3 endoprotocrista:

0. absent
1. present, distal ridge
2. present, forming hypocone

123. P4 protocone:
0. absent or indistinct
1. present, close to paracone in size

124. P4 paraconule:
0. large and distinct
1. small
2. indistinct

125. P4 preparaconule crista orientation:
0. toward parastyle
1. toward paracone

126. P4 metacone:
0. present, distinctly smaller than paracone
1. present, about equal in size to paracone
2. absent

127. P4 metaconule:
0. present, similar in size to paraconule
1. present, significantly smaller than paraconule
2. absent or indistinct

128. P4 metaconule position:
0. distal to line connecting protocone and metacone
1. on line connecting protocone and metacone
2. mesial to line connecting protocone and metacone

129. P4 endoprotocrista:
0. absent
1. present as ridge joined to protocone
2. present, forming hypocone

130. P4 metaloph:
0. absent, no connections between protocone (or hypocone), metaconule, and ectoloph
1. metaconule connects to ectoloph, but not protocone or hypocone
2. metaconule connects to protocone or hypocone, but not ectoloph
3. metaloph complete but low or weak
4. metaloph complete and high

131. P4 hypocone:
0. absent
1. present, weak or poorly separated from protocone
2. present, strong and separate from protocone

132. P3 and P4 metacone position:
0. distal to paracone
1. distolingual to paracone

133. P3-4 cross lophs:
0. not u-shaped
1. u-shaped

134. M1 size:
0. smaller or nearly equal to M2
1. larger than M2

135. M1 ectocingulum (labial cingulum):
0. absent or reduced
1. present but broken at paracone
2. present and continuous

136. M2 ectocingulum (labial cingulum):
0. absent
1. present but broken on paracone
2. present and continuous

137. Upper molar centrocrista:
0. poorly developed
1. present, labially flexed
2. present, not flexed

138. Upper molar mesostyle:
0. absent
1. weak, cingular
2. strong

Comments. This character corresponds to character 7 of Gelfo (2004) which was codified as mesostyle absent (0) in *Simoclaenus* and *Pucanodus*. However, Muizon & Cifelli (2000) describe an incipient mesostyle. Following the later authors, we codify here both taxa as having a weak mesostyle (character state 1).

139. M paracone:
0. not flattened
1. flattened buccally
2. pinched

140. Lingual crest on paracone:
0. absent
1. present

141. M metacone:
0. not flattened
1. flattened buccally
2. part of convex ectoloph

142. M metacone tilting:
0. vertical, in line with paracone
1. metacone lingually tilted

143. M1-2 postmetacrista:
0. weak or absent
1. present and in line with paracone and metacone
2. present and labially deflected

144. M3 postmetacrista:
0. weak or absent
1. present and in line with paracone and metacone
2. present and labially deflected

145. M protocone and hypocone shape:
0. vertical
1. mesially recurved

146. Upper molar paraconules:
0. large and distinct
1. small or indistinct
2. merged into protoloph

147. Upper molar paraconule position:
0. midway between paracone and protocone
1. closer to protocone
2. closer to paracone

148. Upper molar metaconules:
0. present
1. very small
2. absent

149. M metaconules or corresponding part of metalophs:
0. on line between metacone and hypocone
1. mesial to line connecting metacone and hypocone

150. Upper molar parastyles:
0. small
1. large, teardrop-shaped
2. form crest with paracone
3. absent

151. Main mass of M parastyle:
0. in line with paracone and metacone
1. buccal to line connecting paracone and metacone

152. M parastyles:
0. not recurved
1. distally recurved

153. M3 parastyle:
0. similar to that of M1-2
1. projecting buccally

154. M1-2 paracone and metacone size:

0. paracone distinctly larger than metacone
1. paracone and metacone about same size

155. Upper molar preparaconule crista:

0. toward parastyle
1. toward paracone, does not join
2. joined with paracone

156. Upper molar ectoloph-metaloph junction:

0. anterior to metacone, separate
1. anterior to metacone, premetaconule crista bends back to join
2. joins at metacone

157. M1-2 hypocone:

0. absent
1. present

158. M2 posthypocrista:

0. absent or indistinct
1. distinct, short, and mesiobuccally directed
2. distinct, long, and mesially directed
3. distinct, long, mesiobuccally directed, forming basin distal to metaloph

159. M2 lingual cingulum:

0. absent
1. present only as ridge spanning central valley
2. present, except at hypocone
3. present across entire lingual face

160. Labial crest of M hypocone:

0. absent
1. present

161. M3 size:

0. distinctly smaller than M2
1. about same size as M2
2. distinctly larger than M2

162. M3 metacone:

0. similar to that of M2
1. lingually shifted
2. lingually shifted to nearly touching hypocone

163. M3 hypocone:

0. absent
1. present, but distinctly smaller than M3 protocone
2. present, similar in size to M3 protocone

164. **M3 hypocone position:**
0. at about same level as protocone
1. labially shifted relative to protocone
2. lingually shifted relative to protocone

165. **M3 hypostyle:**
0. absent
1. small or narrow cingulum
2. large shelf or cusp
3. posthypocrista continuous with postmetacrista, enclosing basin

166. **M1-2:**
0. square or longer than broad
1. broader than long

167. **Second lower incisor:**
0. not tusk-like
1. enlarged, procumbent, and tusk-like

168. **Distal cusp on i3:**
0. absent
1. present

169. **p1 presence:**
0. present with short diastema
1. present with no diastema
2. absent

170. **Post-p2 diastema:**
0. absent
1. short (less than or equal to p3)
2. long (greater than p3)

171. **p2 paraconid:**
0. absent
1. large and distinct without paralophid
2. present with paralophid
3. paralophid forming loop enclosing mesial basin

172. **p2 metaconid:**
0. absent
1. very small swelling on protoconid slope
2. distinct, small, and distolingual to protoconid
3. large and lingual to protoconid

173. **p2 talonid:**
0. shelf or ridge with no distinct cusps
1. small, medially-placed hypoconid present
2. large, medially-placed hypoconid with well-developed metalophid

3. large, labially-placed hypoconid with well-developed metalophid, entoconid absent
4. large, labially-placed hypoconid with well-developed metalophid, entoconid present

174. p3 metaconid:
0. absent
1. present, close to protoconid
2. present, closer to margin of tooth than to protoconid

175. p3 metaconid size:
0. less than half the height of protoconid (including absent)
1. more than half the height of protoconid but still distinctly smaller
2. about equal in size to protoconid

176. p3 hypoconid:
0. small
1. large
2. absent

177. p3 entoconid:
0. absent
1. present, distinctly smaller than hypoconid
2. present, comparable in size to hypoconid

178. p3 paraconid or paralophid:
0. paraconid not distinct, paralophid no more than short, mesial preprotocristid
1. paraconid distinct cusp, with or without paracristid
2. paralophid well developed without distinct paraconid, defines valley between paralophid and metaconid/protoconid

179. p4 paraconid and paralophid:
0. absent
1. distinct cusp, with or with out paracristid
2. paralophid well developed without paraconid, mesially or mesiolingually directed
3. paralophid well developed without paraconid, extends lingually close to mesial wall

180. p4 width:
0. distinctly narrower than m1
1. as wide or almost as wide as m1
2. wider than m1

Comments. This character was coded as 1/2 for *Mioclaenus*. Zack et al. (2005) codify it as state 1 (p4 markedly larger than m1), but Kondrashov & Lucas (2006) show a *Mioclaenus* specimen in which the p4 is subequal in width to the m1.

181. p4 metaconid: Caracter 19 de Gelfo,

0. absent
1. present, much smaller than protoconid
2. present, about same size as protoconid
3. present, taller than protoconid

182. p4 entoconid:

0. absent or weak
1. present and distinct

183. m1 paraconid or paralophid:

0. distinct, separate paraconid cusp
1. distinct paraconid at lingual end of paralophid appressed to protolophid
2. paralophid extending lingually without distinct paraconid, separate from metaconid
3. paralophid extending lingually and connected to mesial crest from metaconid
4. paralophid extending mesiolingually with valley between it and protolophid
5. short lingually-extending paralophid and labially extending crest from metaconid meeting at mid-protolophid
6. paraconid and paralophid absent or indistinct

184. m3 protolophid shape:

0. straight
1. labial portion angled more distolingually than lingual portion
2. lingual portion angled more distolingually than rest

185. Lower molar protolophid notch:

0. deeply notched nearly to base of cusps
1. shallowly notched to flat

186. Lower molar twinned metaconids:

0. absent
1. present

187. Lower molar protolophid connection to metaconid:

0. protolophid connects to mesial metaconid or between mesial and distal metaconids
1. protolophid connects exclusively to distal metaconid

188. Lower molar metaconid buttress:

0. absent
1. lingual
2. labial

189. Mesial crest of molar metaconid:

0. present
1. absent

190. M metastylids:

0. strong
1. weak
2. absent

191. Lower molar cristid obliqua:
0. oblique, contacts middle of protolophid
1. oblique, contacts lingual cusps
2. longitudinal

192. m2 cristid obliqua shape:
0. straight
1. bowed buccally
2. bowed buccally forming continuous arc with hypolophid
3. bowed lingually

193. m3 cristid obliqua shape:
0. straight
1. bowed buccally
2. forming continuous arc with hypolophid
3. bowed lingually

194. Height of cristid obliqua within valley between trigonid and talonid:
0. cristid obliqua very low or interrupted, valley wide and deep
1. valley filled or reduced by cristid obliqua or encroaching bases of cusps

195. Lower molar talonid height:
0. much lower than trigonid
1. about same height as trigonid

196. m3 hypolophid:
0. incomplete
1. complete, lingual and labial cristids about equal
2. complete, labial cristid longer than lingual

197. m3 hypolophid shape:
0. straight
1. labial portion angled more distolingually than lingual portion
2. slightly concave distally
3. lingual portion angled more distolabially than labial portion

198. Lower molar posthypocristid:
0. present
1. absent

199. Lower molar postentocristid:
0. absent
1. present

200. m3 postentoconulid:
0. absent

1. present, small
2. present, large
3. medial extension of lophoid loop

201. m1 and m2 hypoconulids:

0. large
1. small
2. absent or cingular
3. form enlarged cingular shelf

202. m1 and m2 hypoconulid position:

0. buccal
1. medial
2. lingual

203. m2 hypoconulid:

0. separate from hypolophid
1. closely appressed to hypolophid

204. m3 hypoconulid:

0. present, large
1. small
2. absent or reduced to cingulum

205. m3 hypoconulid position:

0. completely distal of hypoconid and entoconid
1. between hypoconid and entoconid, forming part of hypolophid where present

206. m3 hypoconulid connection:

0. separate
1. joining mid-hypolophid
2. joins postcristid from hypoconid
3. joins postcristid from entoconid

207. Lower molar entoconulid:

0. distinct
1. indistinct or absent

208. Enamel prism decussation:

0. horizontal
1. vertical

Newly added characters

209. Naso-frontal suture on dorsal surface of skull (Missiaen and Gingerich, 2014):

0. wedge or "W"-shaped
1. transverse

210. Reverse alternating carpus (enlarged magnum and thus, lunar-unciform contact is lost, and cuneiform-magnum contact achieved; see Cifelli, 1993):

0. absent
1. present

211. Upper molar paracone, position (Hooker, 1989):

0. not bucally tilted
1. buccally tilted (changing the orientation of pre and postparacristae from longitudinal to oblique)

212. Individualized protostyle on upper molars (Gelfo and Sigé, 2011):

0. absent
1. present

213. Lower molars hypoconids large, extending on the lingual half of the talonid and invades talonid basin anterior to hypoconulid (Muizon and Cifelli, 2000):

0. absent
1. present

214. Enlarged talonid of last lower molar with distinct hypoconulid lobe anteriorly delimited by labial and lingual concavities (Radinsky, 1966; Missiaen and Gingerich, 2014):

0. absent
1. present (the hypoconulid has been posteriorly displaced)

2. Data matrix

Didelphis
0100000000[01]110?000100000000000011020010000000100000000-1-
00000100001120000010000110000000202000000000000002001000-0?00-
00000?????????-00220000000001-2-11011--0-00000-0000000000-200????0010?012---
00001001211001?000000
Asioryctes
0000000001111111-
0100000000000111111010???????????????????0?00000????????000000000?0000?201
000??00?000002000000-0100-0001012-22-100--022010000000002-11000--0-00000--
1001000000000000000000?002---000-100[02]100001?000000
Eotitanops
1200?1?1011110000001?020001010????20020???01210110211?????1?00110?1101101
11112001000011110101 20????????10020100001000-2001011012-
020000001200002201110111011010001 00-
000?000001010[01]21203010?102100111[01]1102--10300101011
Palaeosyops
120101??011110?0001?020001000?11?20020111011101102111010111001101010100
11111200100[02]01112110120111001221002011010 1000-2001011012-
00010000120000220111011101101000100110010000112101312031 10?10111111210
002--10300111011
Orohippus
1100?1??01120??0011?020000010?1112002013?0122112121????11?0011021201001
11112101000111121010211110012210120110 21[01]0121200101001002120002 21200
000000001100010010101020200?102241211221212011021101111020011200021011
1011
Epihippus
1?0001???1?2????????0????????????????0???0????????111????????????1200?0???112
10100?1?1121?10211?110022?01201102120121 2?41010010024200 0??120000220000
110001001000102021??1020422112212140110111111111201111 21[01]031?101011
Mesohippus
100001?10112010001 1?020000010?11?210201300121111202111111111001122120110
1111121010001111210112111110122101201101120121224101001002420002212000
0220000110001001000102021001022422112212141110111110112011112000310101
011
Eohippus
1100?1010112010001 1?020000010?11?200201?00121111212111111111001102120100
1111120010001111211112111110?12210020110211012 12[01]01010010100000022200
0001100001100012110201020 21??00???2?0003121201102120011100001110002101 0
1011
Protorohippus
1100?1??0112010001 1?02000001001 1?200201300121112?21????11?0011021201001
1111200100011112101121 1110?022100201002110121210101001120100002 2210000
11000011000121103010 20210?00001100003121201001110001102001110002101010
11
Xenicohippus
1100?1?1?11211000[01]1?0200000 1??11?20020?300121111212111111111??11021201
001111120010001111210112??????022?00201102010010 2111[01]1101021300002220

000011000011000 1[12]11020102021??1000111000312121110102011110200011000 2
10??1011
Sifrippus
????????????????????????????????????0???0121012?2111??1?????????12010011111
20010001?1111??12?????????????20??????0[01]10[12][01][02][01][01]1011110[13]00
002220000011000011000021102010202 0??????11101312131110[12]01000110-
00101000200??1011
Pliolophus
1100?1?1?11?11??????0?0????????????0??0???0121012?2???????????????120100?????
??????????121?112????????????1201101010121200111011011100002220000011000 01
10001211020102020??10???22001312120110202000110010?1100021???1011
Hyracotherium
1?0??101011111100????0?????????????????1?????????????????????????????????????
???????????????????????????02010000000110200111001111200002212000012000011
000111103010212 0??00????????????2010?2120001100001110002 1?101011
Palaeotherium
1101110001[01]20000011002010001 0?1112102012?1100-
12111110001 1?0011211101011111120010 0[02]00112110120101011221103 01102020
11022012110100222000221210102200020210012012001121200010234221222 12141
110012122111110312100310101011
Plagiolophus
10011101011200000 11?020101010?11?2[01]020???1110-
121111100011?00112?1201011111200100201112101121 1?1--022101301200-
1011002011100100121000221210102200021210012012001220200120011220222121
41110012122111110312000 31?101011
Leptolophus
1001?101011??00??1?00?010101?????20??0??????????????????????????????????????
????????????????????????????1301200-
101100??1111020112100022121010110202121011201200022020??20011210013121 4
111001212211-110?1200031?1?1011
Hallensia
1100?100011100000110020000010?????0020120?12??1????????????001101??01?01?
?112001000?011?????2???10?02210020100??001102011010010001000022010000100
000100001111110101120 0000???1?1???1?13011021200011000011100021 0101011
Propachynolophus
????????????????????????????????????20???????????????????????????????????????
?????????????????????????120100[12]010110213111001011200002 21[01]0000120010
1100012010201021 20??1001021102312020110112100111110011000[13]1???1011
Propalaeotherium
1100?1?1011100000 11?0200000 10?11?200200200121012011 11???11?001102120100
1111120010001111 21??12???10?02210120110[01]010010210111001101[03]0000111
200002200001100010 11[01]30102121001001 12?1?221?12011011210111111011 2000
110??1011
Lambdotherium
1000?1???1?20??0011?0200010[01]0?11?20020????????120?1111111110 0110?1????0
111112001000101121111 2??????0???01201001010000200101 1012-
1200001112000022012111100120101010201100000012010031303111010210011211
13111001001?1011
Homogalax
1100?1???1111??0001?0200000 1??11??0020???01210111211111111?0011021201?01

111120010001?111101120111000221002010010[01]00002001011111001000020200000100111010000211010102201010000021102312020110212200111100011000[12]10
1?1011
Cardiolophus
110001???1111?00001?020000010?11?20020??????????????????????????????????1
120010000?111101120?????????002010[02][01]0[01]00002001011012201000020200
0001101011100012110101020110?00000110003120201102122001111101110002101?
1011
Eomoropus
110??100011100000010020000110?11?22020?????????????????11?00110???01???11
1120010000111210012111100022?01201001010000204101211210400000012211011
10021111112210102021110?2000021002313021110012100112110012110110101011
Litolophus
1100?1???1111??0001?02000?110?11?21020?????2???????????????00110???01??1??1
120010000?1111???201110002210020100101001020410121121040000222021101210
021111102212102021110100001220022130211101021001121100121101101?1011
Moropus
1101010001110000001?020001110?11?220200210010-
1001100110111001111000100111111000000011101001201120-02202120120111000-
2041012112104000000121110111002111111221110112020012020011002212141110
10211111-11002--2--10101011
Lophiodon
1100010?01011??0001?020011010?11?20020??00000-
1211111010011001101000000111112001000001112100120112--
0221013012010100002011012-122030100112001111212-
211001022101011200000200020000221203010?102000111010011100110101011
Isectolophus
1100?1???1111??00?1?02001001??11?20020????????????1111111110011021201??1?
?????????????????????????????0230110201000020310120122040000112000011002-
2110001011010102011??102001100231202110?[12]122001111100101001101?1011
Heptodon
1100010001211000001?020010010011120020??0012101212111???11?001101120110
1111120010000?11211112111100022?0130110[01]-
1000020210121122010000112000012102-
211000021111010202100102011000231202210?102200111000111110110101011
Helaletes
110101?001211000001?020010010?11?20020?30012101212111111111001102120110
1111120010000111211112111100122101301101110000213101211220410001120010
12202-
201000021101011210100102021211231213210?102200111010011110110101011
Lophialetes
110101???1?11??0001?0200??01??11?20020??001220120211????11?001122??011011
1112000000211121111201110112211130110201000020410121121040110112001112
102-211000121101010201101103332201231302210?012200111011111110110101011
Schlosseria
?1???1?????????00????20?????????????20???????????????????1?00112???????111112
000000211121111201110112211130110201000020410121121040110112000112102-
21100012110101020110?103332201231302210?01220011101012--10110101011
Deperetella
????????????????????????????????????0???01120120211001011100112??????011111

21000000111211112011101122100301002120100 2-4121211-1242000002000211012-
201000022103010200 1?1102222212[12]31216010?002---?1111003--2-111??1011
Teleolophus
????????????????????????????????????0??????????????????????????????????????
?????????????????????????013011020201002-4121211-[12]242000002000211012-
2010000221030102011?1102222211[12]31216010?012---?1111003--2-111??1011
Hyrachyus
1100010001111000001002001001 0?11?200201210121012021111101110011021201 10
1111120010010111211112011101122101301102 01000-
2021012112214[01]000112001012102-
20100002110101020110010002121023121201 0?10220011101002--2--11101011
Phenacodus
0100010001001010001002000000101102002011000110011011001011000100011000
01010010011000100000001000001000100201020-
00000100101010000000002202000000000000000010-
112001[01]10000010011000[01]11201-00?100100110-1010111100000000 0
Ectocion
0100?0000101101 0?010020000?????????20???????????1110???????????1????0???0
01001100010?????0?1?????????00201020-0000-
2031010011002000022120000110000111001001100000-
21??010010000[01]11212010?010100110-000011110100?0000
Tetraclaenodon
010001???1011??????00???0??????????0?0???0021001?01111?11000000 0?111100100
00100110300000000100?00?000100201000-0000-[01]001010-
0[01]0002000220[012]0000000000000001--1100000-
21??000001000[01]11201010?100100110-00001111000000000
Meniscotherium
010001000101100000100200001010000200200100011001101000101100000000 11110
010000001110000001001010 0?00?000100201000-[01]00[01]-
[01]1010100[01]0100000000120000200000111001001120000--
1000[01]201000012121[04]110?110122110-00002011210000000
Cambaytherium
1100?1???1011??0011?0200010?????????20?1?0010-
11111011000110010 0?010??0?010120010001111110012?011???21?0011100100000-
1001012-
0110000002200000011000010000000110101121001[01]00010000001[12]05-
0110[01]2---010-[01]02000000001?1110
Nakusia
??
??????????????????????????11???????????????12002-
000000220000001?00001000?0011101?????0???????????????????????????????????????
???????11??
*Anthracobune*_na
??
?????????????????????????111?01?111102??10101100??0000?200000000002013--
0021112010211 0??1011111101022050011012033011210201000 01?1?1110
Anthracobune
???00101
1100000???????????????????111?01?11110[02]??10101[01]00??0000?20000000000 20
13--0021112010211 0??101111110102205001101203301121020100001?1?1110

Pilgrimella
???
?????????????????????????111?0??1111022210101100030000220000000002013--
01211020??2110??1011121111022150011202133010210201000000???1110
Radinskya
1100?1???1011??0001?0?0000010?00?000??????????????????????????????????????
???????????????????????????0201020-
0?????????10010000000220000000000000000010-
1000111000??00??
Phenacolophus
??00111
000???00000201?????????????301?????0??????0-
????????0000?22000000000000011000221120002011?010???2100111202110?1101001
1111003101001???0000
Eritherium
0?1??0?????11???????1?????????????????1??????????????????????????????????????
??????????????????????????0201000-0000-0001112122-100-00221200000001-
2111000001300002130 1?10001001011120601 0?012---?10-1120100001?0?0001
Phosphatherium
0210?01?1121110000111110000 21?????0021?????????????????????????????????????
???????????????????????????10201200-0000-1001012102-0000000200000011 01-
20100012213002020301?20???000001120501 0?012000?1101020000001?000000
Moeritherium
02101000113010000010111000 02??????01211010010-
1100???????????????0011200???1010??1????00010001????????????120120210010-
1001012102-000000000000000001-2-00001-213302021301-
20102221011120?010?012000?1001020100001?000000
Numidotherium
?21??01?11001??0001?11100?02??????0121??00000-
01001?10?011?100???000000?001010101 00000?000211???????????11201200-
0000?0001?12?22-100000000000000001-
200000?2213?020202010200242210101302110?012000?111?000200001?0?0000
Dimaitherium
?????110010110??0010100001????????????1????120-
111??????????????????????????1010100030?0?010212???????????1220110211000-
2001112112-
[12]2[01]000221200001101021110010013202020 30??1021200101111 02010?1120001
1100130100021?0?1001
Microhyrax
????????????????????1??11101
10?301000102 12?????????????20??????????????????????????000120000010122110000
001310002130??100021010001102110?1120001100013211002 1???1001
Saghatherium
010001???1021???????00000?02??????0?211?1?12??12??1?0???1??11000??????????1
01011?030001?102?200?00?0321122011011 2000-1011112002102200000120000 1101-
2111001001300212120001022410001112041 10?11211111111032110021?0?1001
Diacodexis
0000?01001021011-
01002000001[01]100001020?000121012111101111100010000110001101020000000 0

2012?1[01]20??20?0101002010[02]0-0000-0001012022-000-0022200000110000-
000010-0-0-100--1000[01]0000010010000-10?112000110-0100010021?000001
Tiuclaenus
??
???????????????????????201?1?00-0010-10?1012-22-
0000000000000011000010100000000010-
11??10000102011000100000120110102000010000 1???0001
Molinodus
??
???????????????????????101?1?????010-10?1012-22-
0000002220000011000010000000100010-
110?10????????10201010001200001010000100001???0001
Simoclaenus
??????????1???
???????????????????????121?1100-1000-
0??101??????????02201000011000010000000000010-
11??10000?????10201010001201101020100100001???0001
Pucanodus
??
???????????????????????101?1?????010-10?1012-22-
0000001121000011000010000000000010-110-
10000102011010100000120000102000020100 1???0000
Theosodon
0101001?110200?1001001000101?0001?002012101210121 1100-
101101010210201001111120000010011100102010000132 1102?110101000-20?1012-
12-
0000000012101022000010100100100001200001100002 2??020214010011212211101 0
03110121?-11000
Asmithwoodwardia
??
???????????????????????1?1?1?0???010-10?1011012-
0000001120000011000010100000100 0110-
100010000002011020100101120010102000010000 1???1111
Didolodus
??0??01001
00000001??????????????????1?110101011010?1011012-
000000111200001100001000000010001 02010???00001000110201011021200001020 0
0010000[01]???1111
Cramauchenia
110?000??10200??0??00100011 1?0001?0020???012101212?????????????????????????
????????????????????????????122?110201000-10?1012-12-
000000001210 10??000010100100100010201 00??0000?????21214010011212211101 0
03110121?0?1011
Protolipterna
???0011001 11???????????????020100111110
0010010001110002 0?????????001?110????????????12-12-
000000112000001100001000000010000 10-
00??200000020110002011011201001010100100001 00?1011
Thoatherium
0100?00?11020???001001000101?0001?0020121012101212?????????101121020100 1

1111200000100111001020100001321202?110------------12-12-
000000001210102200001010010010001020001-
1000022020212140100112122111010031101 21?111010
Adiantoides
0100?????12111???0100????????????????0???
????????????????????????102?110101000-10?1012-12-
00000000?21010??0000101001001000102001011000022000202040100 11?122111010
03110121?1?1010
Depaulacoutia
???01100110???????????????????????0120
110000001201001 0???????????1?1?0???010-00?1010112-
00000011200000110000100000001000101010???000000???102010100112011010200
011000010??1111
Lamegoia
???01100010????????????????01?????0100
11000000121?00100??????????1?1?0????????????11012-
000000012000001100001000000010002020 10???0000100011120101001120110102 00
001000010??11??
Victorlemoinea
???01100010100-
10100???????????????0100000000011200001 0???????????2?1????????????????????
???????2101022000010100100100010 1000??10000?????20213?10011210?11??10?321
???11??1010
Miguelsoria
?????????????????????????????0001??1100
0100000111 10??20?????????1?1?1?0??????????????????????011200000110000 10000
0001000000-10??2000000011102120010112001010201001000010??1111
Tetramerorhinus
0100000?111200?1001001000101?0001?0020121002101212100-
10110101121020100111112000001001110010201000013 21212?110202000-20?1012-
12-000000001210102200001010010010001020001-
1000022020212140100112122111010031101 21?-11010
Diadiaphorus
0101000?110200?1001001000101?0001?0020121001101212100-
10110101121020100111112000001001110010201000013 21212?110202000-20?1012-
12-000000001210102200001010010010000120001-
1000022020212140100112122111010031101 21?111010
Escribania
???
??????????????????????????1?1???????????????????????????2200001000000110001 0
01000102000????????????????30100002100111210111 11000???1111
Mioclaenus
???
??????????????????????????1?1??????????????12-22-
000000222100001000000100000100000010-
00??10000000000[12]0010000012211012001011100 31???0001
Promioclaenus
???
??????????????????????????1?1?????000-0000012-22-

000001221000001000 1[02]100000100000010-
00??100000010000111000001201101020100110001???0000
Protoselene
???
?????????????????????????1?1?00-??????????10002-
00000122210000110000100001000011020-
00???0???001002001110000120000101000011000 1???0000
Litaletes
???
?????????????????????????1?1?????000-
00010110[02]100000002210000021000010000110000 1010-
00??10100000011021200000120000100000111000 1???0101
Ellipsodon
???
?????????????????????????1?1100-0000-
000001200100000001120000000000110000000000000-
010?2000000000100110000012011010201 02-10011???0100
Choeroclaenus
???
?????????????????????????1?1??????????????12-22-
00000122000000100000100001000000000-
00????????????010010000012011010001001000 01???0000
Orthaspidotherium
0100000101011110101?000100?0??0011102???????????1???1??????????????2???????0
0001101000010101010???????????101000-0000-2001012-1000000002211101022 01-
001100101000010 0-000001011001002021200002120110121000200000 1?0?0000
Pleuraspidotherium
0100000101011 11?101?000100?01?0011102????0120-
1000101000????????1010100?00000011010000101010 10?1???0?????211010-0000-
200101101000000002211101022 01-001100101001011 0-
000022???00100212120000012001012101022121 31?0?0000

3. Synapomorphies of selected nodes.

-Pan-Perissodactyla (Kollpaniidae + ((Didolodontidae + (Litopterna + Perissodactyla)):

115(1). Parastyle of P3 not protruding, mesial edge convex.

118(1). P3 metacone present and smaller than paracone.

165(1). M3 hypostyle small or narrow cingulum.

174(1). p3 metaconid present and close to protoconid.

182(0). p4 entoconid absent.

203(0). m2 hypoconulid separate from hypolophid.

-Didolodontidae + (Litopterna + Perissodactyla):

126(1). P4 metacone present, about equal in size to paracone.

161(1-2). M3 size subequal or larger than M2.

162(1). M3 metacone lingually shifted.

163(1). M3 hypocone present (but smaller than protocone).

188(1). Lower molar metaconid buttress lingual.

211(1). Upper molar paracone buccally tilted.

213(1). Lower molars hypoconids large, extending on the lingual half of the talonid and invades talonid basin anterior to hypoconulid.

-Litopterna + Perissodactyla:

86(1). Navicular facet of astragalus saddle-shaped.

115(0). P3 parastyle protruding, with mesial edge concave.

154(1). M1-2 paracone and metacone about the same size.

179(2). p4 paralophid well developed without paraconid, mesially or mesiolingually directed.

183(3). m1 paralophid extending lingually and connected to mesial crest from metaconid.

191(1). Lower molar cristid obliqua obliquely oriented and contacting lingual cusps.

194(1). Height of cristid obliqua within valley between trigonid and talonid with valley filled or reduced by cristid obliqua or encroaching bases of cusps.

196(1). m3 hypolophid complete, lingual and labial cristids about equal in size.

198(1). Lower molar posthypocristid absent.

203(1). m2 hypoconulid closely appressed to hypolophid.

-*Escribania* + Cambaytheriidae + Anthracobunidae:

188(0). Lower molar metaconid buttress absent.

207(0). Lower molar entoconulid distinct.

212(1). Individualized protostyle on upper molars.

-Perissodactyla:

63(1). First metacarpal absent.

75(1). Presence of astragalar canal.

88(2). Orientation of astragalar facet of calcaneum in lateral view perpendicular to long axis.

128(2). P4 metaconule position mesial to line connecting protocone and metacone.

130(4). P4 metaloph complete but weak.

155(2) Upper molar preparaconule crista joined with paracone.

159(1) M2 lingual cingulum present only as ridge spanning central valley.

197(1). m3 hypolophid with labial portion angled more distolingually than lingual portion.

206(1). m3 hypoconulid connection joining mid-hypolophid.

4. The eight Most Parsimonious Trees (MPTs) that resulted from the phylogenetic analysis.

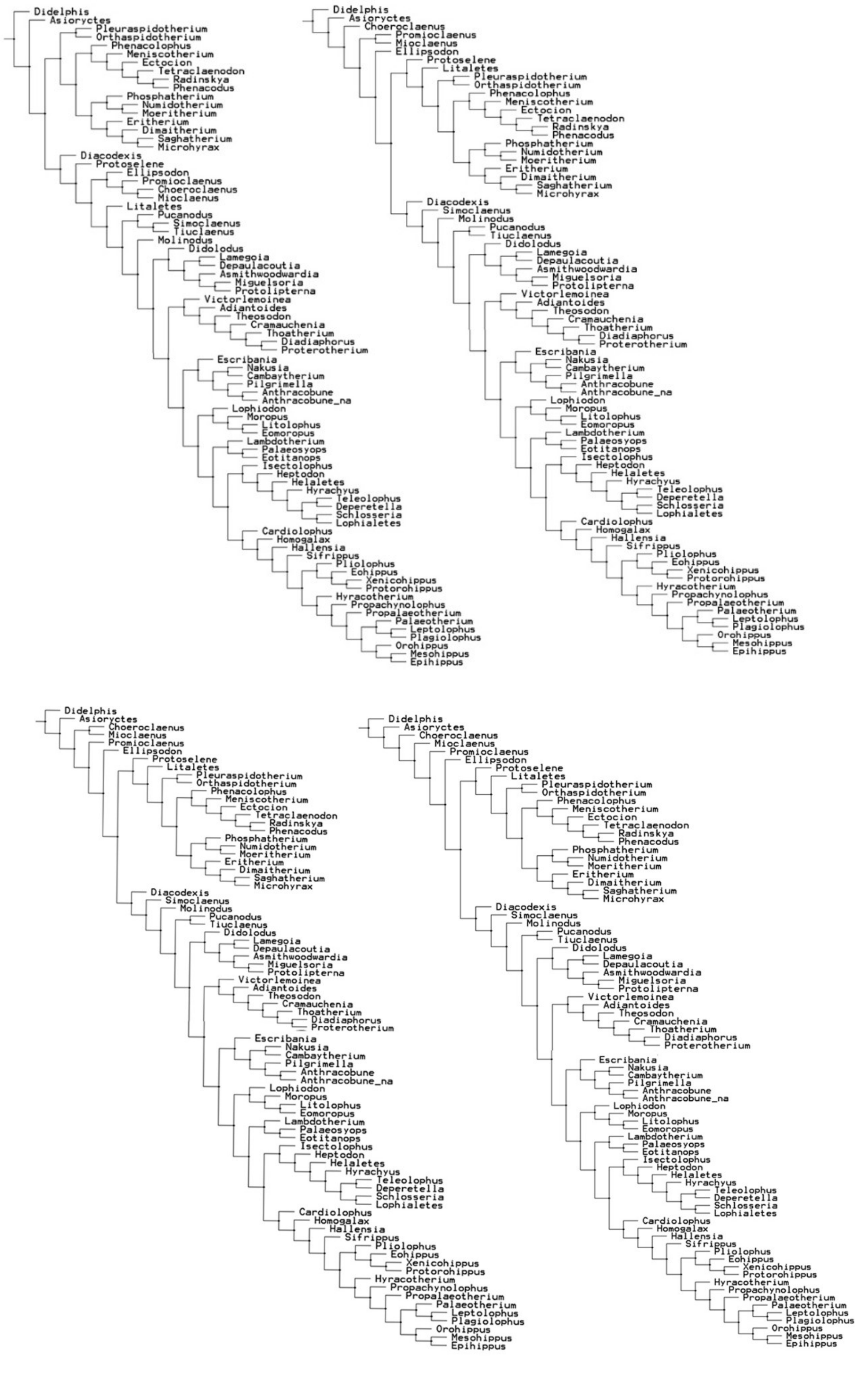

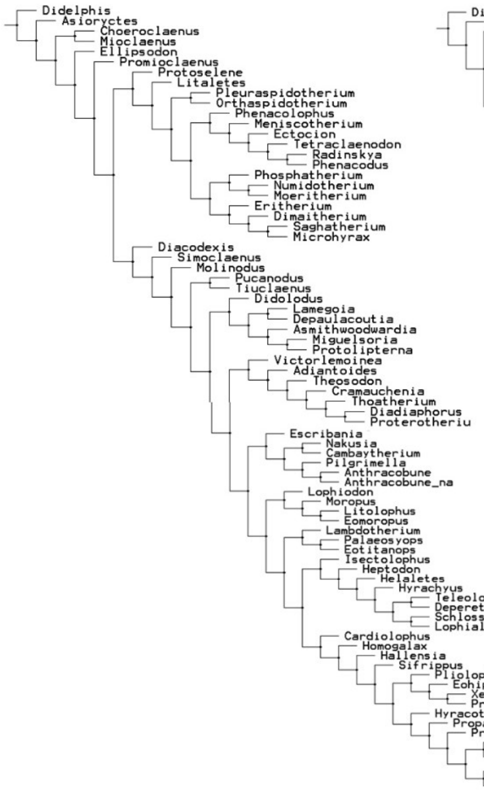
Didelphis
Asioryctes
Choeroclaenus
Mioclaenus
Ellipsodon
Promioclaenus
Protoselene
Litaletes
Pleuraspidotherium
Orthaspidotherium
Phenacolophus
Meniscotherium
Ectocion
Tetraclaenodon
Radinskya
Phenacodus
Phosphatherium
Numidotherium
Moeritherium
Eritherium
Dimaitherium
Saghatherium
Microhyrax
Diacodexis
Simoclaenus
Molinodus
Pucanodus
Tiuclaenus
Didolodus
Lamegoia
Depaulacoutia
Asmithwoodwardia
Miguelsoria
Protolipterna
Victorlemoinea
Adiantoides
Theosodon
Cramauchenia
Thoatherium
Diadiaphorus
Proterotheriu
Escribania
Nakusia
Cambaytherium
Pilgrimella
Anthracobune
Anthracobune_na
Lophiodon
Moropus
Litolophus
Eomoropus
Lambdotherium
Palaeosyops
Eotitanops
Isectolophus
Heptodon
Helaletes
Hyrachyus
Teleolophus
Deperetella
Schlosseria
Lophialetes
Cardiolophus
Homogalax
Hallensia
Sifrippus
Pliolophus
Eohippus
Xenicohippus
Protorohippus
Hyracotherium
Propachynolophus
Propalaeotherium
Palaeotherium
Leptolophus
Plagiolophus
Orohippus
Mesohippus
Epihippus

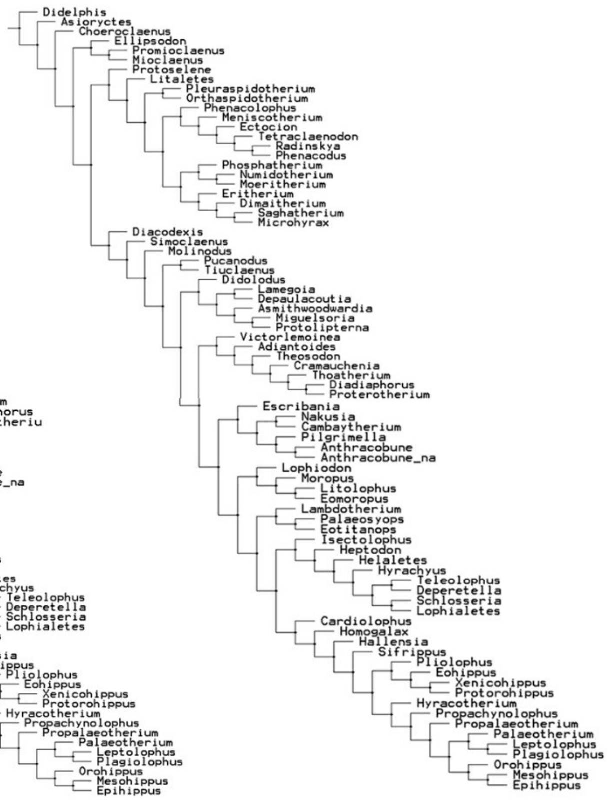
Didelphis
Asioryctes
Choeroclaenus
Ellipsodon
Promioclaenus
Mioclaenus
Protoselene
Litaletes
Pleuraspidotherium
Orthaspidotherium
Phenacolophus
Meniscotherium
Ectocion
Tetraclaenodon
Radinskya
Phenacodus
Phosphatherium
Numidotherium
Moeritherium
Eritherium
Dimaitherium
Saghatherium
Microhyrax
Diacodexis
Simoclaenus
Molinodus
Pucanodus
Tiuclaenus
Didolodus
Lamegoia
Depaulacoutia
Asmithwoodwardia
Miguelsoria
Protolipterna
Victorlemoinea
Adiantoides
Theosodon
Cramauchenia
Thoatherium
Diadiaphorus
Proterotherium
Escribania
Nakusia
Cambaytherium
Pilgrimella
Anthracobune
Anthracobune_na
Lophiodon
Moropus
Litolophus
Eomoropus
Lambdotherium
Palaeosyops
Eotitanops
Isectolophus
Heptodon
Helaletes
Hyrachyus
Teleolophus
Deperetella
Schlosseria
Lophialetes
Cardiolophus
Homogalax
Hallensia
Sifrippus
Pliolophus
Eohippus
Xenicohippus
Protorohippus
Hyracotherium
Propachynolophus
Propalaeotherium
Palaeotherium
Leptolophus
Plagiolophus
Orohippus
Mesohippus
Epihippus

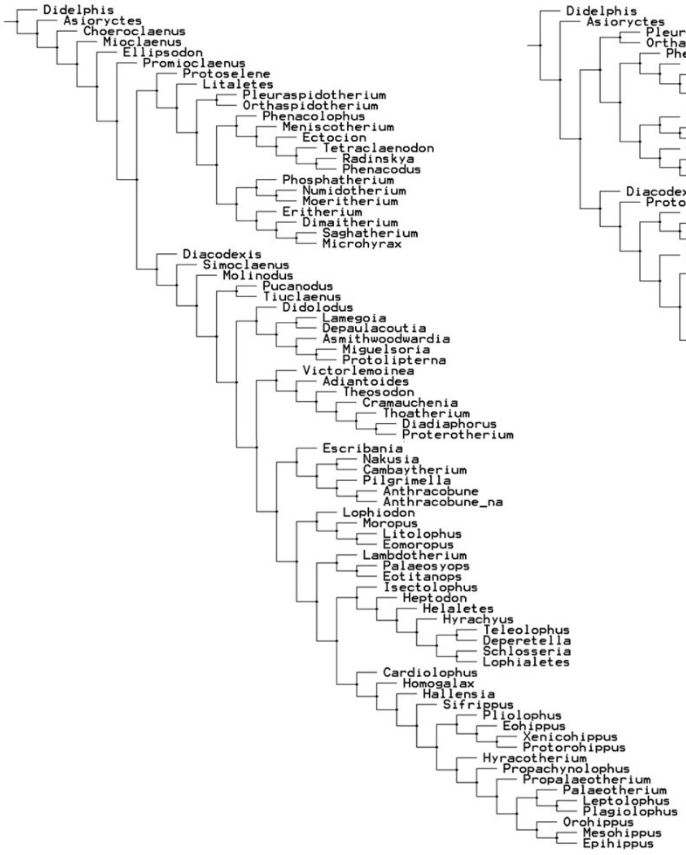
Didelphis
Asioryctes
Choeroclaenus
Mioclaenus
Ellipsodon
Promioclaenus
Protoselene
Litaletes
Pleuraspidotherium
Orthaspidotherium
Phenacolophus
Meniscotherium
Ectocion
Tetraclaenodon
Radinskya
Phenacodus
Phosphatherium
Numidotherium
Moeritherium
Eritherium
Dimaitherium
Saghatherium
Microhyrax
Diacodexis
Simoclaenus
Molinodus
Pucanodus
Tiuclaenus
Didolodus
Lamegoia
Depaulacoutia
Asmithwoodwardia
Miguelsoria
Protolipterna
Victorlemoinea
Adiantoides
Theosodon
Cramauchenia
Thoatherium
Diadiaphorus
Proterotherium
Escribania
Nakusia
Cambaytherium
Pilgrimella
Anthracobune
Anthracobune_na
Lophiodon
Moropus
Litolophus
Eomoropus
Lambdotherium
Palaeosyops
Eotitanops
Isectolophus
Heptodon
Helaletes
Hyrachyus
Teleolophus
Deperetella
Schlosseria
Lophialetes
Cardiolophus
Homogalax
Hallensia
Sifrippus
Pliolophus
Eohippus
Xenicohippus
Protorohippus
Hyracotherium
Propachynolophus
Propalaeotherium
Palaeotherium
Leptolophus
Plagiolophus
Orohippus
Mesohippus
Epihippus

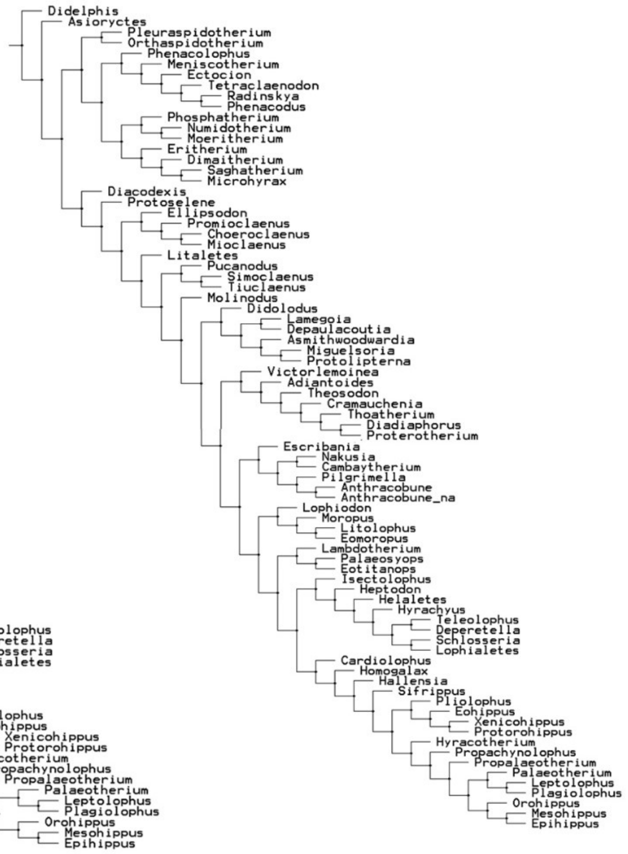
Didelphis
Asioryctes
Pleuraspidotherium
Orthaspidotherium
Phenacolophus
Meniscotherium
Ectocion
Tetraclaenodon
Radinskya
Phenacodus
Phosphatherium
Numidotherium
Moeritherium
Eritherium
Dimaitherium
Saghatherium
Microhyrax
Diacodexis
Protoselene
Ellipsodon
Promioclaenus
Choeroclaenus
Mioclaenus
Litaletes
Pucanodus
Simoclaenus
Tiuclaenus
Molinodus
Didolodus
Lamegoia
Depaulacoutia
Asmithwoodwardia
Miguelsoria
Protolipterna
Victorlemoinea
Adiantoides
Theosodon
Cramauchenia
Thoatherium
Diadiaphorus
Proterotherium
Escribania
Nakusia
Cambaytherium
Pilgrimella
Anthracobune
Anthracobune_na
Lophiodon
Moropus
Litolophus
Eomoropus
Lambdotherium
Palaeosyops
Eotitanops
Isectolophus
Heptodon
Helaletes
Hyrachyus
Teleolophus
Deperetella
Schlosseria
Lophialetes
Cardiolophus
Homogalax
Hallensia
Sifrippus
Pliolophus
Eohippus
Xenicohippus
Protorohippus
Hyracotherium
Propachynolophus
Propalaeotherium
Palaeotherium
Leptolophus
Plagiolophus
Orohippus
Mesohippus
Epihippus

The phylogenetic analysis resulted in 8 Most Parsimonious Trees (MPTs) of a length of 1518 steps. Main differences on each tree almost relay on the interrelationships of taxa that are at the base of the tree. Mioclaenidae, Hyopsodontidae, and related taxa are nested in different trees as stems of Afrotheria, Euungulata, or pan-Perissodactyla, and usually are regarded as para or polyphyletic assemblages. Although not recovered in the strict consensus tree, in the 8 MPTs, *Diacodexis* (assigned as one of the most basal Artiodactyla) recovers as a sister group to the pan-Perissodactyla clade, in agreement with recent molecular analyzes. Kollpaniidae is some trees constitute a monophyetic assemblage, whereas in other cases constitute sucessive sister groups of pan-Perissodactyla. This unstable condition of the tree base is probably due to the poor knowledge of most “condylarths” (most of them only known by dentary pieces or even isolated teeth), but also to some degree of convergence at the base of placental mammal tree. On the other side, on each MPT, the clade including Didolodontidae, Litopterna and Perissodactyla is robust and no important changes on the main topology of the clade are observed.

5. Figure showing phylogenetic results, including branch support measures. From left to right: Bremer support, absolute bootstrap frequency, GC bootstrap frequency.

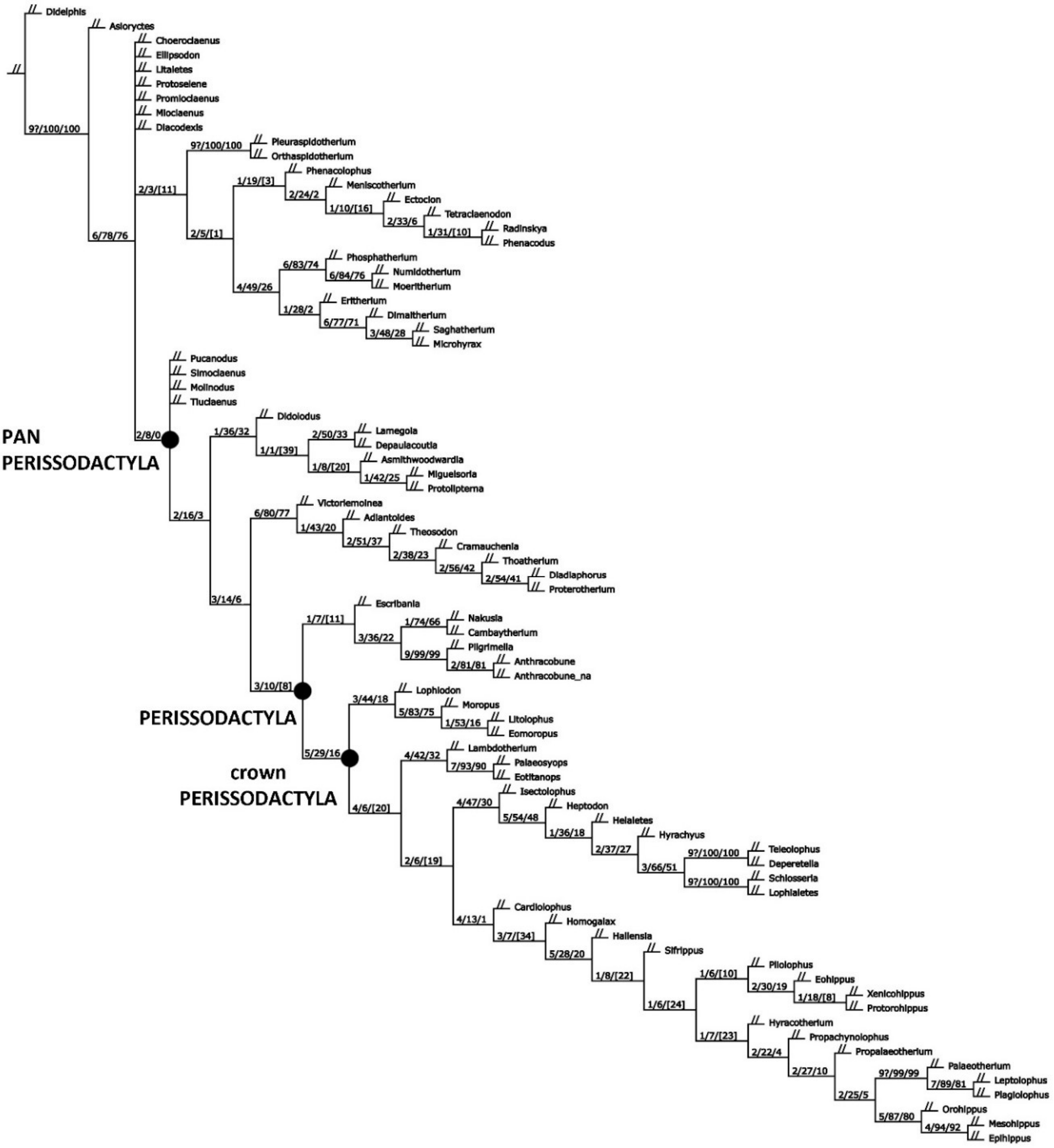

Supplement: Supplementary file 1 — Supplementary file1 (PDF 1347 kb) [file 41598_2020_70287_MOESM1_ESM.pdf]
